# Impact of High-Risk Sex and Focused Interventions in Heterosexual HIV Epidemics: A Systematic Review of Mathematical Models

**DOI:** 10.1371/journal.pone.0050691

**Published:** 2012-11-30

**Authors:** Sharmistha Mishra, Richard Steen, Antonio Gerbase, Ying-Ru Lo, Marie-Claude Boily

**Affiliations:** 1 Department of Infectious Disease Epidemiology, Imperial College, London, United Kingdom; 2 St. Michael’s Hospital, University of Toronto, Toronto, Canada; 3 Department of Public Health, Erasmus MC, Rotterdam, The Netherlands; 4 World Health Organization, Geneva, Switzerland; University of Toronto, Canada

## Abstract

**Background:**

The core-group theory of sexually transmitted infections suggests that targeting prevention to high-risk groups (HRG) could be very effective. We aimed to quantify the contribution of heterosexual HRGs and the potential impact of focused interventions to HIV transmission in the wider community.

**Methods:**

We systematically identified studies published between 1980 and 2011. Studies were included if they used dynamical models of heterosexual HIV transmission, incorporated behavioural heterogeneity in risk, and provided at least one of the following primary estimates in the wider community (a) the population attributable fraction (PAF) of HIV infections due to HRGs, or (b) the number per capita or fraction of HIV infections averted, or change in HIV prevalence/incidence due to focused interventions.

**Findings:**

Of 267 selected articles, 22 were included. Four studies measured the PAF, and 20 studies measured intervention impact across 265 scenarios. In low-prevalence epidemics (≤5% HIV prevalence), the estimated impact of sex-worker interventions in the absence of risk compensation included: 6–100% infections averted; 0.9–6.2 HIV infections averted per 100,000 adults; 11–94% and 4–47% relative reduction in prevalence and incidence respectively. In high-prevalence epidemics (>5% HIV prevalence), sex-worker interventions were estimated to avert 6.8–40% of HIV infections and up to 564 HIV infections per 100,000 adults, and reduce HIV prevalence and incidence by 13–27% and 2–14% respectively. In both types of epidemics, greater heterogeneity in HIV risk was associated with a larger impact on the fraction of HIV infections averted and relative reduction in HIV incidence.

**Conclusion:**

Focused interventions, as estimated by mathematical models, have the potential to reduce HIV transmission in the wider community across low- and high-prevalence regions. However, considerable variability exists in estimated impact, suggesting that a targeted approach to HIV prevention should be tailored to local epidemiological context.

## Introduction

The concept of behavioural heterogeneity and core-groups has been critical to our understanding of how sexually transmitted infections (STI) are transmitted and persist in a population [1], [2], [3]. Core-group theory implies that a relative few individuals are more likely to become infected and disproportionately more likely to transmit infections, such that in the absence of this heterogeneity the STI epidemic could fail to establish and persist [2], [4]. It has therefore been suggested that epidemic control could be better achieved by focusing interventions on high-risk groups (HRGs) [5].

Behavioural heterogeneity often reflects the presence of a HRG that consists of individuals who engage in multiple serial or concurrent partnerships at a frequency greater than the rest of the population. Sex work is one such example. Women and men who sell sex have a larger number of sexual partners and in some cases, due to social marginalization, they have less access to treatment or even condoms to reduce infectivity [2]. Therefore, transmission is high within commercial partnerships. Over time, infection moves from this ‘core-group’ to a wider population through a ‘bridge’ population [2], [6]. Commonly, this bridge comprises men who have sex with sex workers and non-commercial partners. For heterosexual STI epidemics, other sources of heterogeneity include circular migration because of the potential for associated changes in partnerships as individuals travel back and forth between home and destination (for example, seasonal short-term migration for work) [7], [8], [9], casual multiple partnerships, and other concurrent partnerships outside of commercial sex.

Some countries have adopted a focused approach to HIV prevention, and are targeting interventions to HRGs [5], [10]. But in other countries, particularly in sub-Saharan Africa, only a few regions are targeting interventions to HRGs such as female sex workers (FSWs) [11], [12]. Recent data suggests that HIV prevalence among FSWs in Kenya and Uganda exceed 40% [13], [14], [15]. Earlier in the epidemic, more than 80% of FSWs working in Nairobi were infected with HIV [16], [17]. A paucity of data on the presence and size of HRGs remains an obstacle to delineating the role of HRGs in high-prevalence epidemics and to implementing focused interventions in many regions of the world [18]. Insights into the contribution of HRGs, particularly FSWs and clients, to different HIV epidemics could help to inform HIV prevention strategies [19].

By simulating counterfactuals (i.e. “what-if” scenarios), mathematical models provide a platform for the assessment of (a) the contribution of HRGs to HIV transmission in the wider community (herein referred to as “overall transmission”), and (b) the population-level impact of any one or a combination of behavioural, biological, and structural interventions focused on HRGs. In this study, we systematically review published dynamic mathematical modelling studies of heterosexual HIV transmission which measured the contribution of HRGs or the potential impact of focused interventions. First, we summarize the model features, populations, and time-horizon of impact assessment. We then explore the potential sources of variability in model estimates across simulations and present pooled estimates where possible.

## Methods

We searched PubMed, MEDLINE, and EMBASE for studies published between 1980 and 31 December, 2011. The search included the following terms: (HIV* or AIDS [MeSH term or abstract]) and (model*[keyword or abstract]) and (in all fields, [“math*” or “transmission” or “dynamic*” or “stochastic” or “compartment*” or “deterministic” or “agent-based” or (“agent based”) or “individual-based” or (“individual based”) or” network*” or “simulation*” or (“computer simulation*”) or “micro-simulation*” or “discrete-time” or (“discrete time”) or (“discrete-event*”) or “discrete event*”]). There were no language restrictions. Following the removal of duplicates, all titles and abstracts were screened for exclusion. When a citation was considered potentially relevant or the title/abstract was deemed insufficient for a decision on inclusion or exclusion, the full text of the article (and online supporting material or appendix) was evaluated. One reviewer (SM) conducted the search and data extraction.

### Definitions and Inclusion/exclusion Criteria

A HRG consisted of one or more of the following subgroups: FSWs; clients; men and women with casual or long-term multiple (serial or concurrent) partnerships; circular migration or in-migration associated with commercial sex, or with multiple partnerships. A non-commercial high-risk group was restricted to men and women with casual or long-term multiple partnerships.

Studies were included if they used dynamical models of heterosexual HIV transmission, incorporated behavioural heterogeneity in HIV risk between individuals, and quantified the population impact of either of the following primary outcomes: (1) HIV transmission from a HRG to the wider community, or (2) interventions focused on a HRG. For the first outcome, studies were included if they measured the cumulative population attributable fraction over t years (PAF_t_) of transmitted events due to a HRG (fraction of incident infections in the wider community that would fail to manifest in the absence of transmission within and from a HRG). For the second outcome (intervention impact), studies were included if they measured the absolute number or fraction of new infections prevented (prevented fraction, PF), or the relative change in HIV prevalence/incidence over any time-period. All types and combinations of focused interventions were considered. Wherever possible, we extracted outcomes measured in the total population (TP, including the HRG). Outcomes in the general population (GP, total population excluding the HRG) or in the female GP were used if the first indicator was not available.

Mathematical models that did not incorporate a dynamical relationship between prevalence and incidence, such as cohort and static models were excluded. We excluded reviews without primary modeling results, models in conference abstracts alone, and unpublished studies, because their methodology and results could not be comprehensively assessed.

To avoid confusion with the various definitions and use of the terms ‘concentrated’ and ‘generalized’ epidemics [20], [21], we divided epidemic size into high-prevalence (current or endemic HIV prevalence in the total or general population, or female GP, >5%) and low-prevalence (current or endemic HIV prevalence in the total or general population, or female GP, ≤5%).

### Exploratory Analysis of Study Results for Sources of Variability in Model Estimates

A single modeling study often includes outcomes from multiple scenarios. Within a single study, scenarios could vary with respect to epidemiologic and intervention-related assumptions. In order to quantify the sources of variability in model outcomes, we examined all scenarios (N_s_) within each study (N). For each scenario, we extracted primary outcomes, epidemiologic characteristics, and intervention-related assumptions.

We summarized the different outcomes across studies in forest plots stratified by epidemic characteristics, which allowed for a visual assessment of sources of variability. If there were at least 15 scenarios that measured a primary outcome, we performed an exploratory (hypothesis generating) analysis to describe influential sources of variability in outcomes. First we assessed the univariate fraction of variance explained by epidemiologic and intervention-related assumptions. If a covariate explained >10% of the variability in a given outcome, we explored its relative influence on outcomes using the partial correlation coefficient across scenarios which included FSWs and clients. The exploratory analysis was performed separately for low- and high-prevalence epidemics, and was restricted to the following primary outcomes: relative reduction in HIV incidence, PF, and number of HIV infections averted (for high-prevalence epidemics), due to focused interventions. The following available covariates were considered (table 1): epidemiologic characteristics (overall HIV prevalence, epidemic phase, HIV prevalence in subgroups, ratio of HIV prevalence between subgroups, type of HRG (FSW; FSW and clients; FSW, client and non-commercial HRG; non-commercial HRG), ratio of partner exchange rates between subgroups [risk differential]); and intervention-related parameters (prevention tool, coverage of HRG, efficacy in reducing HIV susceptibility per sex act [or transmission probability if intervention effect on HIV susceptibility was not differentiated from intervention effect on HIV infectivity], time-horizon for outcome measurement). Table 1 lists the covariates which varied within studies.

**Table 1:** Intervention impact and covariates examined in the exploratory analysis for sources of variability in model outcomes.

	HIV infections averted per100,000 adults	Fraction of HIV infections averted		Relative change in HIV incidence	
Overall HIV prevalence	>5%	≤5%	>5%	≤5%	>5%
N studies (of 20 which measured impact of focused intervention)	4	6	4	4	4
N_s_ scenarios (of 265** which measured the impact of focused intervention)	52	100	38	20	36
**Covariates examined**					
**Epidemiologic characteristics**					
Overall HIV prevalence	√	√*	√*	√*	√*
Ratio of HIV prevalence among FSWs to general population females		√*	√*	√*	
Ratio of HIV prevalence among clients to general population males		√*			√*
Ratio of number of clients to high-risk females		√	√	√	
Size of the FSW population (% of total adult females)		√	√	√*	√*
HIV prevalence among FSWs		√*	√*	√*	
HIV prevalence among clients		√*			√*
Risk differential among females: ratio of yearly partner exchange rate(FSW to general population females)		√	√	√	√*
Late phase compared withgrowth phase (reference group)		√*	√*	√	√*
**Intervention-related characteristics**					
Prevention tool		*			*
Condom use	√	√	√	√	√
STI treatment	√	√	√		
Condom use & STI treatment	√	√	√		√
Oral pre-exposure prophylaxis	√		√		
Oral pre-exposure prophylaxis & condom use	√				
Vaginal microbicide		√	√	√	√
Vaccine	√		√	√	
Structural intervention (sexual violence)					√
Intervention coverage of high-risk group	√*	√*	√*	√*	√
Intervention efficacy***	√*	√	√	√	√
Time-horizon for outcome measurement (years)	√	√	√	√*	√
Risk compensation versus no risk compensation	√*	√	√	√	√
**High-risk group (HRG)**	*				
FSWs	√	√	√	√	√
FSWs and clients	√	√	√		√
FSWs, clients, and non-commercial HRG	√	√	√		
Non-commercial HRG					√

* Covariate varied within studies (as well as between studies).

** Number of scenarios (N_s_) from the 20 studies (N), include scenarios which measured the relative change in HIV prevalence (N_s_ = 7), and the number of infections averted in low-prevalence epidemics (N_s_ = 12). STI (sexually transmitted infection). FSW (female sex worker). Non-commercial HRG refers to individuals who engage in multiple (non-commercial) partnerships.

*** Efficacy in reducing HIV susceptibility per sex act (or transmission probability if intervention effect on HIV susceptibility was not differentiated from intervention effect on HIV infectivity).

We defined epidemic phase as ‘late’ if primary studies classified their epidemic phase as ‘late’, ‘mature’, ‘plateau’, ‘peak’, ‘stable’, ‘endemic’, ‘endemic equilibrium’, or the HIV prevalence was either declining or stable at the time of outcome measurement. All other time points were classified as the ‘growth’ phase. Because the primary study outcomes could be measured in different populations (TP, GP, or female GP), analyses were adjusted for this covariate.

We summarized primary outcomes by (a) tabulating the estimated range across epidemic size, intervention-related model assumptions, and type of HRG, and (b) pooling point estimates from simulated scenarios for homogenous subgroups. Because most studies did not provide an uncertainty range (or variance) around point estimates for each simulated scenario, the pooled estimates were un-weighted. Results are stratified by low- and high-prevalence epidemics, unless otherwise stated. The analysis was performed in Stata version 11 (StataCorp.).

## Results

### Characteristics of Studies

**Figure 1:**
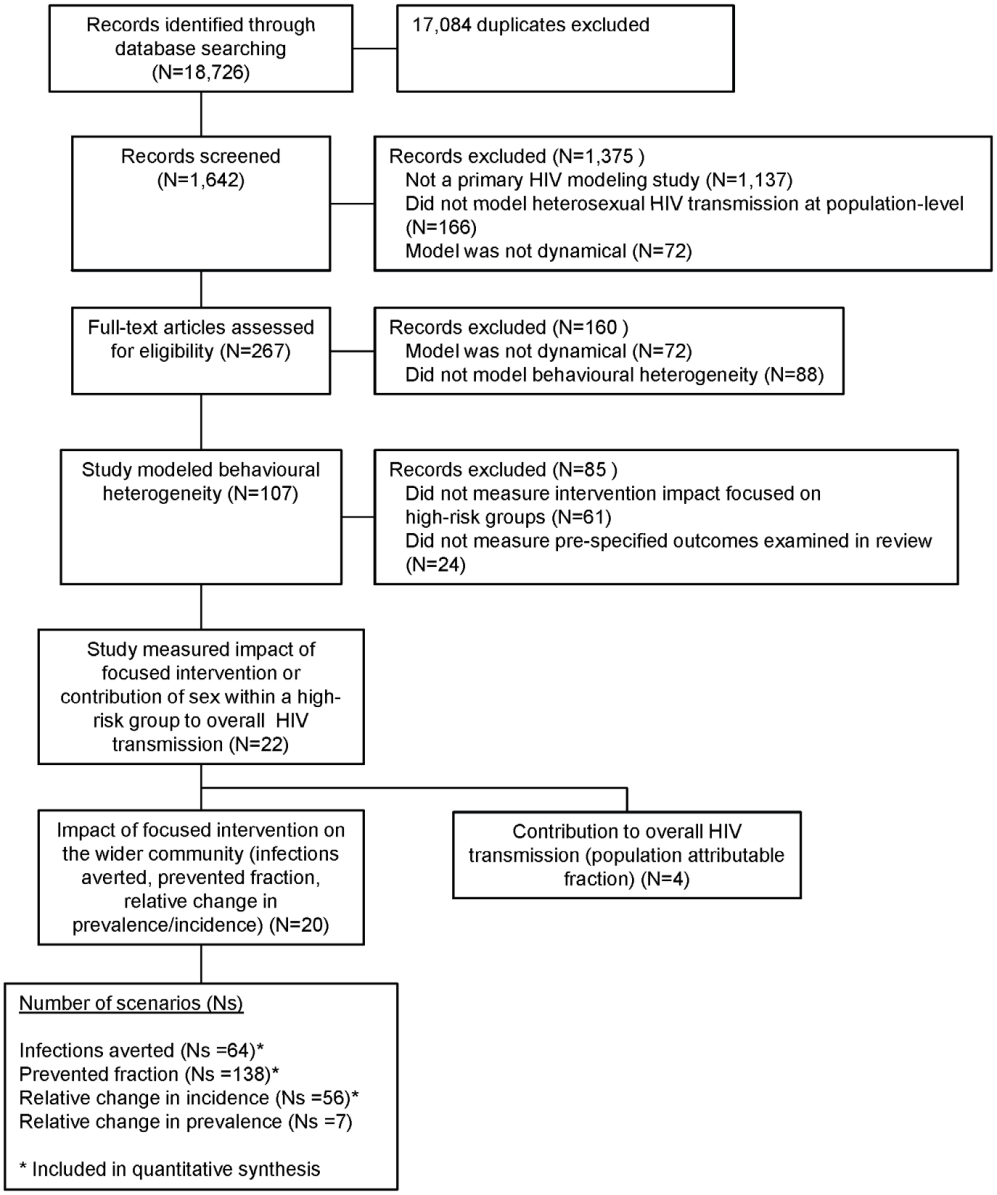
Results of search (PRISMA flow diagram) [43] **.** N refers to the number of studies. Among the studies that measured the outcomes of interest, some could fall into more than 1 category. Note that a total of 144 studies were excluded because models were not dynamic.

Our search criteria identified 18,726 citations, of which 1,642 were unique records (figure 1). Of 267 selected articles, 22 studies were included for analysis.

Table 2 summarizes the main model features of included studies. Additional details are provided in the supplementary tables (table S1, table S2). Most models were deterministic (N = 21), and parameterized with region-specific epidemiological and behavioural data (N = 19). Eleven models were calibrated to observed HIV prevalence and used a single baseline parameter set. The individual-based model analyzed different plausible epidemics that agreed with observed HIV prevalence trends, but were generated using one set of parameters [22]. Thus, this study took account of random fluctuations in an epidemic rather than parameter uncertainty [22]. Four studies employed a random search of the parameter space to identify multiple parameter sets that reproduced (“fit”) observed HIV prevalence data [23], [24], [25], [26]. These four studies conducted an uncertainty analysis after fitting, whereas 13 of the remaining studies conducted a sensitivity analysis (varying parameters without refitting to data) to measure the influence of behavioural, epidemiological, or intervention parameters (table 2).

**Table 2:** Characteristics of included studies.

Study	Setting, high-risk group	HIV prevalence* % (population)	Model characteristics					Outcome of interest
			Model	HIV stage	Parameterization			
					Regional	Fitting or calibration	Sensitivity/uncertainty analysis	
Nagelkerke 2011 [35]	Thailand (SW)	1.7 (total pop.)	Det.	√	√	√	√ (S)	FI
Hontelez 2011 [22]	South Africa (multiple partnerships)	30 (total pop.)	Ind.	√B	√	√	√ (S)	FI
Vickerman 2010 [23]	South India (SW)	0.4–1.3 (total females)	Det.	√B	√	√ (M)	√ (U)	Contribution FI
Xiridou 2010 [29]	The Netherlands (migrants withmultiple partnerships)	0.2 (total pop.)	Det.	√	√			Contribution
Watts 2010 [46]	Non-regional (sexual violence)	0.5–2.5% per year (incidence, total pop.)	Det.				√ (S)	FI
Rao 2009 [34]	India (SW)	0.36 (total pop.)	Det.	B	√	√	√ (S)	FI
Lopman 2009 [28]	Zimbabwe (widows with multiple partnerships)	8–28 (low-risk females)	Det.	√	√			Contribution
Hallet 2008 [37] **	Zimbabwe (SW)	21–24 (total pop.)	Det.	√B	√	√	√ (S)	FI
Boily 2008 [32]	South India (SW)	0.3–4.8 (total females)	Det.	√B	√	√	√ (S)	FI
Deering 2008 [27]	South India (migrants and SW)	1.2–3.2 (low-risk females)	Det.	√	√	√	√ (S)	Contribution FI
Vissers 2008 [36]	Botswana, south India, Kenya (SW)	22, 1.3, 16 (low-risk pop.)	Det.	√	√	√	√ (S)	FI
Johnson 2007 [38]	South Africa (SW)	26 (low-risk pop.)	Det.	√B	√	√	√ (S)	FI
Abbas 2007	Sub-Saharan Africa (SW and multiple partnerships)	20 (total pop.)	Det.	√	√	√	√ (S)	FI
Williams 2006 [24]	South India (SW)	0.7–2.2 (low-risk females)	Det.	√B	√	√ (M)	√ (U)	FI
Vickerman 2006 [26] **	South Africa (SW)	27 (low-risk females)	Det.	√B	√	√ (M)	√ (U)	FI
Vickernan 2006 [25]	South Africa, Benin (SW)	30, 3.3 (ANC)	Det.	√B	√	√ (M)	√ (U)	FI
Nagelkerke 2002 [33]	Botswana, India (SW)	30, 5 (total pop.)	Det.	√B	√	√	√ (S)	FI
Boily 2002 [30] **	Benin, Sub-Saharan Africa (SW)	1–9 (low-risk females)	Det.	√B	√	√	√ (S)	FI
Kakehashi 1998 [31]	Japan (SW)	1.8 (total female pop.)	Det., pair		√			FI
Boily 1997 [47]	Non-regional (SW)	70 (total pop.)	Det.					FI
Kault 1995 [48] **	Non-regional (multiple partners)	0.1–10 (total pop.)	Det.	B				FI
Anderson 1995 [49]	Sub-Saharan Africa (multiple partners)	20–25 (low-risk females)	Det.	√	√		√ (S)	FI

SW (commercial sex work [female sex worker and clients]); B(Co-factor effect of a sexually transmitted infection); FI (focused intervention); (M) multiple parameter sets that are calibrated or fitted to observed data; S (sensitivity analysis using single calibrated/fitted parameter set); U (uncertainty analysis using multiple calibrated/fitted parameter sets); Sensitivity and uncertainty analysis refer to an examination of the influence of parameter values on estimated outcome. Type of model (deterministic [det.], individual-based models [ind]). Outcome of interest specifies the outcomes measured in the study (impact of a FI and/or the contribution of a high-risk group or behaviour to overall transmission). Migrant refers to short-term or circular migration.

* HIV prevalence prior to implementing the focused intervention or at the time from which contribution of HRGs was assessed; if different time-points were assessed, then HIV prevalence reflects the most recent estimate. pop. (population).

** Studies which examined different epidemic phases.

The 22 studies focused primarily on epidemics in south India and selected countries in sub-Saharan Africa (table S3). Commercial sex interventions (in isolation or as part of interventions targeted to non-commercial HRGs) were examined in 11 low-prevalence epidemics, and in 9 high-prevalence epidemics (table 2).

### Contribution of HRG to Overall HIV Transmission: PAF

Four studies measured the contribution of HRGs across 11 scenarios and over different time-horizons (figure 1, figure S1, table S1). Therefore, we did not pursue further analysis to examine the sources of variability in model estimates. In south India, the estimated PAF_1_ of commercial sex (sex between FSWs and clients) ranged between 86.4–97.5% in males and 12–42% in females (N = 1) [23]. In the same region, the estimated contribution of short-term client migration to overall transmission in the total population was 50% over 34 years (PAF_34_) compared to 99% over 44 years (PAF_44_) under the assumption that local sex work remained constant [27]. In other words, another male took the place of a client who periodically left home [27]. In Zimbabwe, the PAF_20_ of widowhood (assuming widows had a higher HIV prevalence than the wider population and/or engaged in multiple partnerships) ranged between 8 and 17% [28].

The contribution of migration to overall transmission was complex, and was influenced by assumptions about the sexual behaviour of migrants at home and away, as well as the sexual behaviour of non-migrants while their partners were away. In the Netherlands, if individuals who immigrated from high-prevalence regions also engaged in high-risk sex locally, the PAF_1_ of in-migration ranged between 22–53% [29].

### Impact of Focused Intervention

Across 20 studies (figure 1), 265 scenarios examined a focused intervention and measured the following outcomes: relative change in incidence (N_s_ = 56); relative change in prevalence (N_S_ = 7); prevented fraction (N_s_ = 138); or number of infections averted per 100,000 adults (N_s_ = 64). The time-horizon for outcome measurement ranged from one year to an endemic equilibrium which could take, in general, more than 20 years to achieve (figure S2, figure S3, figure S4, figure S5).

Intervention impact varied considerably between studies (figure S2, figure S3, figure S4, figure S5). The range of model estimates (in the absence of risk compensation) is presented in tables 2 and 3, and summarized across HRGs. In low-prevalence epidemics (table 3), interventions targeted to FSWs in the absence of risk compensation, were estimated to achieve the following: a PF between 6–97% over 1–11 years [23], [24], [27], [30] up to 100% (local elimination) in the long-term [31]; a relative reduction in HIV prevalence between 11–94% after 3–30 years [30], [32], [33], [34]; a relative reduction in HIV incidence between 4–47% after 1–10 years [23], [32], [35]; and the prevention of 0.9–6.2 HIV infections per 100,000 adults per year over 9 years [36].

**Table 3:** Range of intervention impact, by outcomes measured in the wider community for epidemics with an HIV prevalence ≤5%, in the absence of risk compensation.

	HIV infections averted per 100,000 adults*	Fraction of HIV infections averted	Relative change in HIV prevalence	Relative change in HIV incidence
**N studies**	1 [^36^]	4 [23], [24], [27], [30], [31]	5 [30], [32], [33], [34], [48]	3 [23], [32], [35]
**Epidemic phase**				
Growth	–	7 to 100%^αγ^	23 to 94%↓^αγ^	–
Late	0.9 to 6.2^γ^	6 to 50%^αβ^	11 to 40%↓^βγω^	4 to 47%↓^βγ^
**Type of focused intervention (range of** **% efficacy, range of % coverage)**				
Condom use (87–100, 20–100)	–	13 to 100%^αβγ^	11 to 94%↓^αβγω^	4 to 47%↓^β^
Condom use & STI treatment (70–100, 50–85)	–	6 to 97%^α^	14 to 87%↓^αγ^	–
Oral PREP (50–90, 25–50)	0.9 to 6^γ^	–	–	–
Oral PREP (50–90, 25–50), condom use (100,92.5)	2.7 to 6.2^γ^	–	–	–
HIV vaccine** (78,60)	–	–	–	16.4 to 22.1%↓^γ^
Anti-retroviral treatment (100,50)	–	–	23%↓^γ^	–
**Coverage of high-risk group**				
<60%	0.9 to 3.3^γ^	7 to 40^αβγ^	–	16.4 to 22.1%↓^γ^
≥60%	3.3 to 6.2^γ^	6 to 100%^αγ^	11 to 94%↓^αβγω^	4 to 47%↓^β^
Intervention efficacy**				
<60%	0.9 to 3.3^γ^	13 to 16%^β^	–	4 to 22%↓^β^
≥60%	3.3 to 6.2^γ^	6 to 100%^α^	11 to 94%↓^αβγω^	16.4 to 47%↓^γβ^
**Time-horizon (years)**				
1	–	6 to 40%^α^	–	4 to 15%↓^β^
2–9	0.9 to 6.2^γ^	7 to 25%^αβ^	11 to 34↓^βγω^	5 to 47%↓^β^
10	–	14 to 97%^α^	40 to 78%↓^αγ^	16.4 to 22.1%↓^γ^
>10	–	20 to 100%^αγ^	25 to 94%↓^αγ^	–
**High-risk group (HRG)**				
FSWs	0.9 to 4.6^γ^	6 to 100%^αβγ^	11 to 14%↓^β^	4 to 47%↓^βγ^
FSWs and clients	1.8 to 6.2^γ^	9 to 98%^αβ^	–	
FSWs, clients, and non-commercial HRG	–	22 to 86%^α^	–	
Non-commercial HRG	–	–	40 to 66%↓^γ^	–

Population in which the outcome was measured includes: ^α^low-risk females; ^β^general population (excludes high-risk groups); ^γ^total population (includes high-risk groups); ^ω^ante-natal clinic attendees. Infections averted refer to the following: * per 100,000 uninfected adults (Vissers 2008 [36]). ** Efficacy in reducing HIV susceptibility per sex act (or transmission probability if intervention effect on HIV susceptibility was not differentiated from intervention effect on HIV infectivity).N = number of studies. STI (sexually transmitted infection). PREP (pre-exposure prophylaxis). ↓(decline). FSW (female sex worker). Non-commercial HRG refers to individuals who engage in multiple (non-commercial) partnerships.

In high-prevalence epidemics (table 4), interventions targeted to FSWs in the absence of risk compensation, were estimated to avert 6.8–40% of new HIV infections over 20 years [30]; reduce HIV prevalence by 13–27% over 10–30 years [30], [33]; and reduce HIV incidence by 2–14% over 1 year [26]. Two models estimated that 10 to 564 HIV infections could be averted per 100,000 adults per year in high-prevalence epidemics following a commercial sex intervention [26], [36].

**Table 4:** Range of intervention impact, by outcomes measured in the wider community for epidemics with an HIV prevalence >5%, in the absence of risk compensation.

	HIV infections averted per 100,000 adults	Fraction of HIV infections averted	Relative changein HIV prevalence	Relative change in HIV incidence
**N studies**	3 [26], [36], [39]	3 [22], [30], [39]	5 [30], [33], [47], [48], [49]	3 [26], [37], [46]
**Epidemic phase**				
Growth	–	9 to 48%^α^	19 to 75%↓^αγ^	2 to 65%↓^γ^
Late	10 to 14,617^γ*#^	0.8 to 28.8%^γ^	5 to 11%↓^γ^	10 to 35%↓^γ^
**Type of focused intervention (range of** **% efficacy, % coverage)**				
Condom use (100, 20–100)	10 to 14^γ#^	–	27 to 75%↓^γ^	–
STI treatment (50–100,20)	21 t o 44^γ#^	–	–	14%↓^γ^
Condom use & STI treatment (50–100, 20–100)	41 to 65^γ#^	9 to 48%^α^	19%↓^α^	10 to 50%↓^γ^
Vaginal microbicide (45,75)	–	–	–	–
Oral PREP (50–90, 25–50)	26 to 14,617^γ*#^	0.8 to 28.8%^γ^	–	–
Oral PREP (50–90, 25–50), condom use (100,62.5)	235 to 909^γ*^	–	–	–
Vaccine (78**, 100)	–	5 to 18%^γ^	5 to 11%↓^γ^	–
Anti-retroviral treatment (100,50)	–	–	13%↓^γ^	–
Partner reduction° (N/A,100)	–	–	8%↓^γ^	–
Structural intervention (sexual violence) (100,100)	–	–	–	2 to 65%↓^γ^
**Coverage of high-risk group**				
<60%	10 to 419^γ*#^	0.8 to 30%^αγ^	5 to 19%↓^αγ^	13 to 14%↓^γ^
≥60%	251 to 14,617^γ*#^	5 to 48%^αγ^	8 to 75%↓^γ^	2 to 65%↓^γ^
Intervention efficacy**				
<60%	26 to 419^γ*#^	0.8 to 6.8%^γ^	5 to 11%↓^γ^	–
≥60%	10 to 14,617^γ*#^	5 to 48%^αγ^	8 to 75%↓^αγ^	2 to 65%↓^γ^
**Time-horizon (years)**				
1	10 to 65^γ#^	9 to 30%^α^	–	14%↓^γ^
2–9	26 to 909^γ*^	–	8%↓^γ^	10 to 50%↓^γ^
10	5,356 to 14,617^γ#^	0.8 to 48%^αγ^	5 to 75%↓^αγ^	–
>10		9 to 30%^α^	13 to 27%↓^γ^	2 to 65%↓^γ^
**High-risk group (HRG)**				
FSWs	10 to 564^γ*#^	6.8 to 40%^α^	13 to 27↓^αγ^	2 to 14%↓^γ^
FSWs and clients	159 to 909^γ*^	9 to 48%^α^	–	10 to 50%↓^γ^
FSWs, clients, and non-commercial HRG	5,356 to 14,617^γ#^	0.8 to 28%^γ^	–	–
Non-commercial HRG	–	–	5 to 75%↓^γ^	2 to 65%↓^γ^

Population in which the outcome was measured includes: ^α^low-risk females; ^β^general population (excludes high-risk groups); ^γ^total population (includes high-risk groups); ^ω^ante-natal clinic attendees. °Reduce partnership rate per year (among clients 20 to 15; among FSWs 400 to 50). **Efficacy in reducing HIV susceptibility per sex act (or transmission probability if intervention effect on HIV susceptibility was not differentiated from intervention effect on HIV infectivity). Infections averted refer to the following: *per 100,000 uninfected adults per year of intervention (Vissers 2008 [36]); (^#^per 100,000 adults per year of intervention (Abbas 2008 [39] and Vickerman 2006 [26]_ENREF_43_ENREF_43). N = number of studies. STI (sexually transmitted infection). PREP (pre-exposure prophylaxis). ↓(decline). FSW (female sex worker). Non-commercial HRG refers to individuals who engage in multiple (non-commercial) partnerships.

**Figure 2:**
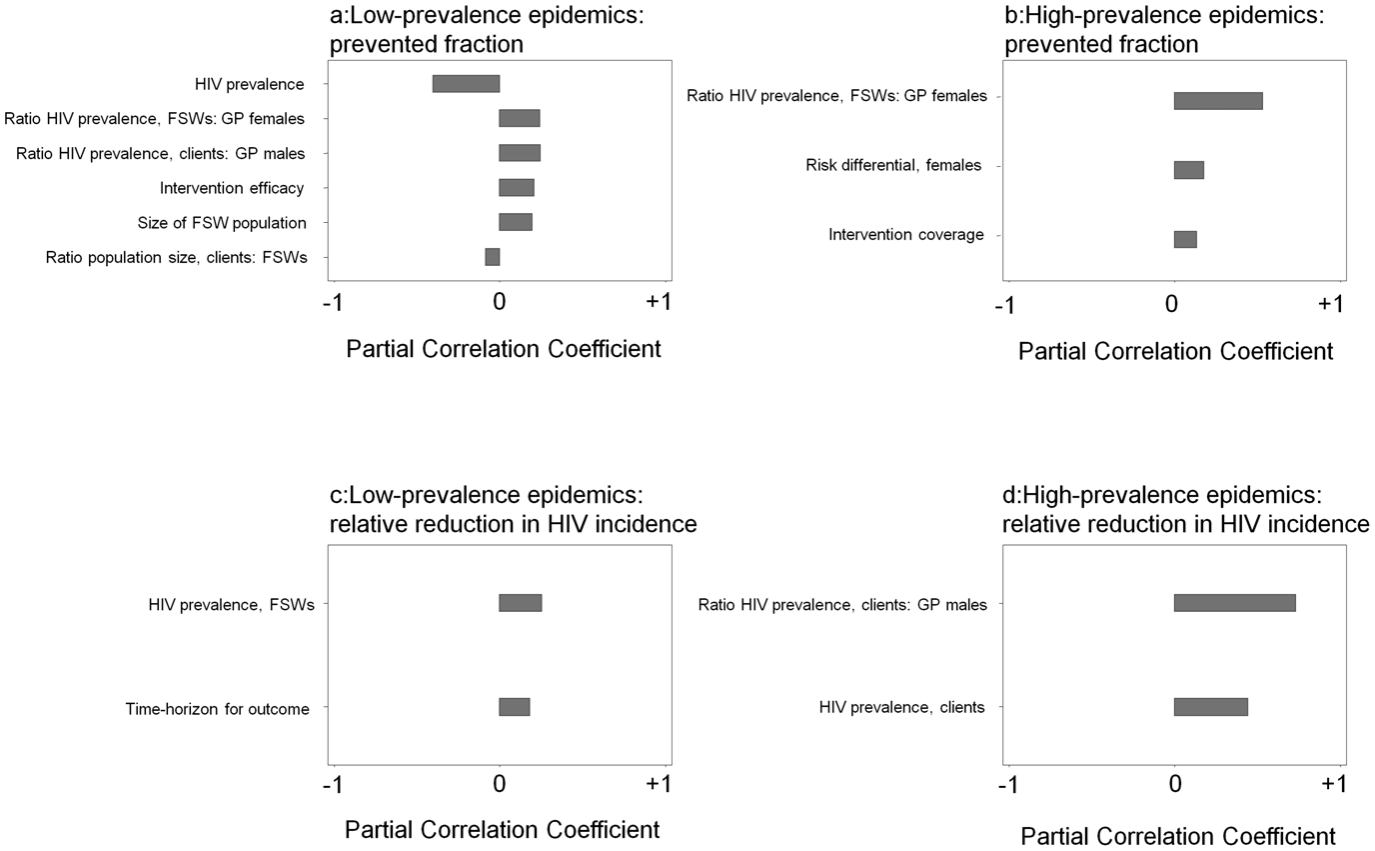
Tornado plot of the partial rank correlation coefficients. The coefficients range between −1 to +1, and indicate the relative influence (and direction) of epidemiologic and intervention-related characteristics in contributing to the variability in model outcomes. Model outcomes include: (a) the fraction of HIV infections averted in low-prevalence epidemics (≤5%) following a focused intervention; (b) the fraction of HIV infections averted in high-prevalence epidemics (>5%) following a focused intervention; (c) the relative reduction in HIV incidence in low-prevalence epidemics (≤5%) following a focused intervention; (d) the relative reduction in HIV incidence in high-prevalence epidemics (>5%) following a focused intervention. All scenarios included FSWs and clients. FSW (female sex worker); GP (general population, does not include high-risk groups). Efficacy refers to the % reduction in HIV susceptibility per sex act.

After stratifying by epidemic size and outcome, univariate assessment of the fraction of variance explained by epidemiologic and intervention-related characteristics (table S4), and subsequent ranking of the partial correlation coefficients (figure 2), revealed important sources of variability in model outcomes.

### Sources of Variability: Epidemiologic Characteristics

Epidemiologic characteristics were important sources of variability for interventions simulated in both low- and high-prevalence epidemics (table S4, figure 2).

In low-prevalence epidemics, the following epidemiologic characteristics were influential sources of variability in the estimated PF: the ratio of HIV prevalence in FSWs to general population females, the ratio of HIV prevalence in clients to general population males, size of the FSW population, ratio of clients to FSW population size, and overall HIV prevalence (figure 2a). In high-prevalence epidemics, a larger HIV prevalence ratio between FSWs and GP females, and a larger risk differential in partnership rates between FSWs and GP females were correlated with a greater impact (figure 2b). The ratios of HIV prevalence or partnership rates between FSWs and GP females provide a proxy for the level of heterogeneity in the population. Across epidemic size, a larger ratio was associated with a larger PF (figure 2a–b).

Intervention impact on the PF was attenuated as overall HIV prevalence increased in the low-prevalence epidemics (figure 2a). This variability in intervention impact across geographically ‘similar’ epidemics was also observed within studies that examined more than one district using the same model in south India [23], [24]. The same FSW intervention (treatment for STIs and condom-use) was estimated to avert 20–25% of HIV infections in Mysore (HIV prevalence in the female GP, 0.7%), but 10–12% of infections in Bagalkot (HIV prevalence in the female GP, 2.2%) over 5 years [24].

Four studies compared the impact of a focused intervention in different countries. The findings highlight how the type of outcome modifies the relationship between intervention impact and epidemic size. STI-based interventions achieved a greater impact in Benin (9% PF) as compared with a high-prevalence scenario (19% PF in sub-Saharan Africa) [30]. The reduction in HIV incidence after the introduction of a vaginal microbicide was greater in Benin than in South Africa (27 to 29% relative reduction versus 2.2 to 11.5% over 4 years) [25]. The same condom-based intervention was estimated to achieve a 3-fold greater reduction in overall HIV prevalence in India than in Botswana [33]. However, as illustrated with oral PREP in commercial partnerships (tables 2–3, figure S3), the absolute number of new infections averted per capita was 100-fold greater in larger epidemics [36]. In the absence of risk compensation, the model estimated that for every 100,000 uninfected adults per year, 0.9–6.2 HIV infections could be averted in India over 9 years [36]. Yet the same intervention was estimated to avert between 26–909 and 44–831 HIV infections per 100,000 uninfected adults per year in Botswana and Kenya, respectively [36].

### Sources of Variability: Intervention-related Characteristics

Variability in the type of prevention tool was not an important source of heterogeneity in outcomes. Based on this analysis, intervention coverage and efficacy were more influential than the type of prevention tool (table S4).

In addition, the time-horizon for outcome measurement was an important source of variability in estimated impact (table S4, figure 2c). For example, in Benin, a program which could increase condom use from 50 to 60% and treat 50% of gonorrhoea infections in FSWs was estimated to prevent 22% of HIV infections in 1 year, but 85% of HIV infections over 10 years [30]. A focused intervention that increased condom use from 20–45% to 80–100% and decreased bacterial STIs by 10% among FSWs, was estimated to reduce HIV incidence by 10% after 5 years and by 35% after 10 years [37]. Intervention impact increased over time because the direct prevention of a single case of HIV by the intervention aborted all secondary transmission events that would have taken place from that one case.

Risk compensation (modeled as an increase in risk-taking behaviour among individuals who received an intervention) was not an influential source of variability across scenarios (table S4), but was influential within studies. Risk compensation was examined for HIV vaccines, vaginal microbicides, and oral PREP [25], [35], [36], [38], [39]. In a study examining oral PREP in India, if FSWs and clients using PREP decreased condom use from 90% to 75%, the impact was attenuated from 6 fewer infections (no risk compensation) to 17 more infections per 100,000 uninfected adults [36]. In the case of HIV vaccines, 200 additional infections per 100,000 vaccinated adults were estimated to occur in the presence of a 25–50% reduction in condom use if vaccines were (a) less effective at reducing infectivity, and (b) provided to FSWs without pre-screening for HIV [38]. None of the models examined risk compensation behaviour in the wider population as a result of an intervention driven decrease in overall HIV prevalence, which could potentially have a larger unwanted impact.

### Impact of Focused Interventions: Summary Estimates

While the exploratory analysis yielded noteworthy patterns, other potential factors, such as the nuances of interventions (frequency of STI treatment, waning efficacy), were too few in number to examine. Epidemiologic characteristics such as the level of mixing between high- and low-risk groups were difficult to standardize across studies. Summary estimates were therefore limited to low-prevalence scenarios that measured the PF following interventions targeted to FSWs (table 5), and could be pooled across intervention efficacy in the absence of risk compensation. In low-prevalence epidemics, FSW interventions with >60% efficacy were estimated to avert a median of 20.5% (range, 7–43%) of HIV infections in the wider community over the short-term (≤5 years, table 5, N_s_ = 12, N = 3), and a median of 59.0% (range, 40–100%, N_s_ = 47, N = 3 ) in the long-term (>5 years).

**Table 5:** Summary estimates of the fraction of infections prevented in the wider population, when interventions were focused on FSWs, in the absence of risk compensation.

	Median prevented fraction (range, N_s_), HIV prevalence ≤5%	
	Time-horizon for outcome assessment ≤5 years	Time-horizon for outcome assessment >5 years
Intervention efficacy≥60%*	20.5% (7 to 43, N_s_ = 12^α^) [24], [30]	59.0% (40 to 100, N_s_ = 47^αγ^) [27], [30], [31]

Population in which the outcome was measured includes: ^α^low-risk females; ^β^general population (excludes high-risk groups); ^γ^total population (includes high-risk groups); ^ω^ante-natal clinic attendees.STI (sexually transmitted infection). N_s_ refers to the number of simulated scenarios. Summary estimates reflect the median and range if ≥5 scenarios across at least 2 studies were available within each category. Estimates were grouped across intervention-related characteristics. *Efficacy in reducing HIV susceptibility per sex act (or transmission probability if intervention effect on HIV susceptibility was not differentiated from intervention effect on HIV infectivity). FSW (female sex worker).

## Discussion

A small number of modeling studies demonstrated that interventions focused on HRGs, particularly FSWs, could be effective, even in high-prevalence epidemics. Notable themes emerged from this analysis, including influential sources of variability in reported outcomes across scenarios and studies. Our review identified significant gaps in the literature: a limited scope of epidemic types and regions have been explored to date, both in the measurement of the contribution of HRGs and the impact of focused interventions in the wider community. There was insufficient information to derive summary estimates of the contribution of specific HRGs to overall transmission, to recommend one prevention tool over another, or to compare the relative impact of focusing interventions on a given HRG versus another. Nonetheless, our findings demonstrate key insights for HIV prevention policies and the design of intervention programs.

### Factors which Influence the Estimated Impact of a Focused Intervention

First, intervention impact was influenced by intervention efficacy and coverage, and importantly, the time-horizon for outcome assessment. Even with the rapid scale-up of a focused intervention, it takes time to observe the impact of preventing secondary or indirect transmission events (infections among persons who did not directly receive the intervention) [30], [37]. As a result, short-term evaluations by HIV prevention programmes may underestimate the impact of focused interventions [30], [37].

Second, within each given focused intervention explored, the estimated impact varied considerably by epidemiologic context. Even within low-prevalence scenarios, and after grouping according to intervention-related characteristics, the PF varied by nearly 50%. Residual variability was due in part to epidemiologic characteristics (such as the difference in partnership rates and HIV prevalence between subgroups, size of the FSW population, and baseline HIV prevalence). Hence, even after stratifying by epidemic size, the estimated impact of a focused intervention is likely to vary by differences in epidemiologic characteristics. This finding has important policy implications. Categorizing regions by overall HIV prevalence alone [20] will be insufficient for making decisions about whether or not to prioritize focused interventions. Generalizability of model outcomes from one region to another will depend on the extent to which (a) we understand the HIV epidemic in the latter, and (b) the two regions have similar epidemiologic characteristics. Empirical data confirms how variable regions are with respect to heterogeneity in HIV risk [15] as well as the size and behavior of sex worker populations [18], [40]. The relative risk of HIV in FSWs (compared with females in the general population) range from 3.4 to 67.4 in sub-Saharan Africa, and from 0.8 to 54.3 in Asia [15]. Hence, the findings from this review suggest that focused interventions should be tailored to the local epidemiologic context.

Third, interventions targeted to FSWs were effective in both low- and high-prevalence epidemics. When the prevented fraction was measured, intervention impact was larger in low-prevalence scenarios. In contrast, the per capita infections averted was 100-fold greater in magnitude in sub-Saharan Africa compared with India, although this important insight was obtained from only one study [36]. The issue of FSW (with or without client) interventions in high-prevalence epidemics is important [19]. At present, sex worker interventions are not widely applied in most high-prevalence countries [11], [12], [41]. The findings from this review suggest that interventions focused on FSWs could have an important role to play in high-prevalence (often called ‘generalized’) epidemics.

As policy-makers design prevention policies in response to their local HIV epidemic, these three issues will be important to consider, in addition to the pragmatic issues of identifying and reaching HRGs.

### Knowledge Gaps

However, if we are to tailor a combination of interventions according to local epidemiological characteristics, it will be critical to understand the potential role of focused interventions across a larger range of epidemics, prevention tools, and HRGs. The contribution of HRGs, particularly FSWs and clients [19], and the preventive potential of focused interventions remains under-researched in high-prevalence settings. Additional studies (and within-study scenarios) of prevention tools are required to estimate the comparative effectiveness of different intervention packages across epidemiologic context, and by focusing on different HRGs. The potential impact of targeting HRGs for anti-retroviral treatment [33] as prevention (differentiated from treatment for individual benefit) also requires further study.

The level of evidence garnered from models depends on the objective of the study. Modeling studies that aim to provide qualitative, illustrative, or fundamental insights are distinguished from region-specific, predictive [42] modeling studies that intend to directly guide intervention programming. The latter should include region-specific data for model parameterization, a calibrated or fitted model to observed outcomes, and an uncertainty or sensitivity analysis that examines the influence of parameters on estimated outcomes. We found that 16 studies met the above criteria. However, only 4 recent studies took into account parameter uncertainty by using multiple calibrated parameter sets instead of one baseline parameter set [23], [24], [25], [26]. A single parameter set may lead to overly optimistic or pessimistic estimates, whereas the use of multiple calibrated parameter sets allows for parameter uncertainty by providing a range of plausible outcomes. Multiple fits also enable us to assess which parameters have the greatest influence on estimated outcomes.

Therefore, further study is needed into different combinations of interventions within a targeted approach, in different epidemiologic contexts, focused on different HRGs, and with the use of multiple calibrated parameter sets. Existing and new models could help address these gaps.

### Limitations

To date, there are no standard reporting guidelines for systematic reviews of mathematical modeling studies, and we therefore followed guidelines developed for empirical studies [43]. Previous modeling reviews have been mostly narrative [44], [45]. We have tried to make our review as quantitative as possible, as well as assess study quality. The considerable variability between published models provided important insights in the exploratory analysis, but also limited our ability to derive pooled summary estimates or to provide comparative estimates of intervention impact by type of prevention tool. Pooled estimates were restricted to 2–3 studies for interventions targeted to FSWs. Our findings are also limited because many regions and prevention tools remain under-researched. While we detected a positive correlation between intervention efficacy and coverage, there was insufficient data to suggest which prevention tools were most effective (after controlling for other epidemiologic and intervention-related sources of variability). It is important to note that our analysis for influential sources of variability on model outcomes was exploratory in nature, and was performed similar to a sensitivity analysis. If covariates did not vary considerably between scenarios, they were unlikely to emerge as an important source of variability in model outcomes. We did not explicitly account for within-study correlation of epidemiologic and intervention-related characteristics, because our objective was restricted to a descriptive analysis. Yet as we found in this review, there is a great potential for synthesis of modeling studies. As illustrated in the search matrix (table S3), studies to date preclude a region- or intervention-specific synthesis of HRG contribution or focused intervention impact, especially by different HRGs. As regional and outcome gaps are addressed, a critical and quantitative synthesis will greatly improve our use of model-based evidence. Comparative modeling reviews could become particularly useful and important for policy-makers as different models in different epidemiologic contexts are used to answer the same questions (much like with empirical research).

### Conclusions

Modeling studies demonstrate that short-term evaluations could underestimate the potential impact of interventions prioritized to HRGs. The modeled impact of focused interventions across epidemiologic context underscores the importance of understanding local heterogeneity in HIV risk – a process that requires estimating the size of HRGs (such as FSWs) and their relative risk of HIV. Despite their infrequent application in practice [11], [12], [41], focused interventions (including interventions targeted to FSWs) could be effective in high-prevalence epidemics. Further study is needed into the contribution of HRGs (particularly FSWs and clients) in many under-researched regions with a high burden of HIV.

## Supporting Information

Figure S1 **Contribution of high-risk group (HRG) or behaviours to overall HIV transmission.** Contribution is measured as the population attributable fraction (PAF, %) of new infections due to a HRG. Study estimates (diamond) and/or the range of within-study estimates are shown for different HRGs and by epidemic phase, and by epidemic size (overall HIV prevalence ≤5% [black], and HIV prevalence >5% [blue]).The PAF was measured in the total population with the exception of Vickerman 2010 [23]. FSW (female sex work) and client migration refers to circular migration. *Client migration associated with more sex work (sw) refers to an increase in the number of local men who pay for sex when migrant clients are away. **Widowhood in the context of a high-prevalence epidemic where HIV prevalence among widowed men and women was 54% and 61%, respectively, and widowed individuals engaged in high-risk sexual partnerships (a larger number of partnerships and preferential mixing with non-widows to preferential mixing with other widows) [28].
(PDF)

Figure S2 **Prevented fraction (%) following a focused intervention.** Prevented fraction depicted for various types of intervention, by an aggregate of coverage and efficacy (coverage multiplied by efficacy), and time-horizon for the outcome measurement (years) within studies. Study estimates (diamond) and/or the range of within-study estimates are shown by epidemic size (overall HIV prevalence ≤5% [black], and HIV prevalence >5% [blue]). Efficacy refers to the reduction in HIV susceptibility per sex act (or transmission probability if intervention effect on HIV susceptibility was not differentiated from intervention effect on HIV infectivity). *Risk compensation (Abbas 2007) was modeled as a doubling in the number of partners per year among individuals who received the targeted intervention [39]. *Risk compensation (Vickerman 2006) was modeled as a 5% decrease in baseline condom use (set at 85%) [25]. A vaccine that reduces HIV susceptibility by 78% in the first year with a waning immunity thereafter, repeated every 2 years (**), or every 5 years (***)[22]. STI refers to bacterial sexually transmitted infections.
(PDF)

Figure S3 **The number of HIV infections averted per 100,000 adults per year after the implementation of a focused intervention.** Impact depicted for various types of intervention, by an aggregate of coverage and efficacy (coverage multiplied by efficacy), and time-horizon for the outcome measurement (years) within studies. Study estimates (diamond) and/or the range of within-study estimates are shown by epidemic size (overall HIV prevalence ≤5% [black], and HIV prevalence >5% [blue]). *Presence of risk compensation. Efficacy refers to the reduction in HIV susceptibility per sex act (or transmission probability if intervention effect on HIV susceptibility was not differentiated from intervention effect on HIV infectivity). Commercial sex work includes interventions focused of FSWs or FSWs and clients. The results of a vaccine study are not shown in this forest plot because outcome was measured as infections averted per 100,000 adults who received the intervention [38]. **Vissers 2008 (per 100,000 uninfected adults per year) [36]; Vickerman 2006, Abbas 2007 (per 100,000 adults per year) [26], [39]. STI refers to bacterial sexually transmitted infections.
(PDF)

Figure S4 **Reduction in prevalence (%) following a focused intervention.** Impact depicted for various types of intervention, by an aggregate of coverage and efficacy (coverage multiplied by efficacy), and time-horizon for the outcome measurement (years) within studies. Study estimates (diamond) and/or the range of within-study estimates are shown by epidemic size (overall HIV prevalence ≤5% [black], and HIV prevalence >5% [blue]). Efficacy refers to the reduction in HIV susceptibility per sex act (or transmission probability if intervention effect on HIV susceptibility was not differentiated from intervention effect on HIV infectivity). **Overall prevalence was measured in the antenatal clinic population. STI refers to bacterial sexually transmitted infections. ART refers to combination anti-retroviral treatment.
(PDF)

Figure S5 **Reduction in incidence (%) following a focused intervention.** Impact depicted for various types of intervention, by an aggregate of coverage and efficacy (coverage multiplied by efficacy), and time-horizon for the outcome measurement (years) within studies. Study estimates (diamond) and/or the range of within-study estimates are shown by epidemic size (overall HIV prevalence ≤5% [black], and HIV prevalence >5% [blue]). Efficacy refers to the reduction in HIV susceptibility per sex act (or transmission probability if intervention effect on HIV susceptibility was not differentiated from intervention effect on HIV infectivity). *Risk compensation in Vickerman 2006 was modeled as a decline in condom use from 85% to 80% among those who use the microbicide [25]. Risk compensation (Nagelkerke 2011) modeled as a decline in condom-use from 70% to 50% among those who receive the vaccine [35]. STI refers to bacterial sexually transmitted infections.
(PDF)

Table S1 Summary of contribution of high-risk group or behaviours to overall HIV transmission.
(DOC)

Table S2 Summary of focused intervention impact.
(DOC)

Table S3 Matrix of modeling studies by geographic region, focused intervention, and measured outcomes.
(DOC)

Table S4 Exploratory univariate analysis of the fraction of variance explained by epidemiological and intervention-related assumptions on model estimates.
(DOC)

Text S1 PRISMA checklist.
(DOC)
