# Supplementary material for: Impact of High-Risk Sex and Focused Interventions in Heterosexual HIV Epidemics: A Systematic Review of Mathematical Models
Source: PLoS One. 2012 Nov 30;7(11):e50691. doi: 10.1371/journal.pone.0050691 (PMC3511305; doi:10.1371/journal.pone.0050691)

Figure S1

Population attributable fraction (PAF) of new HIV infections due to high-risk behavior/group (%)

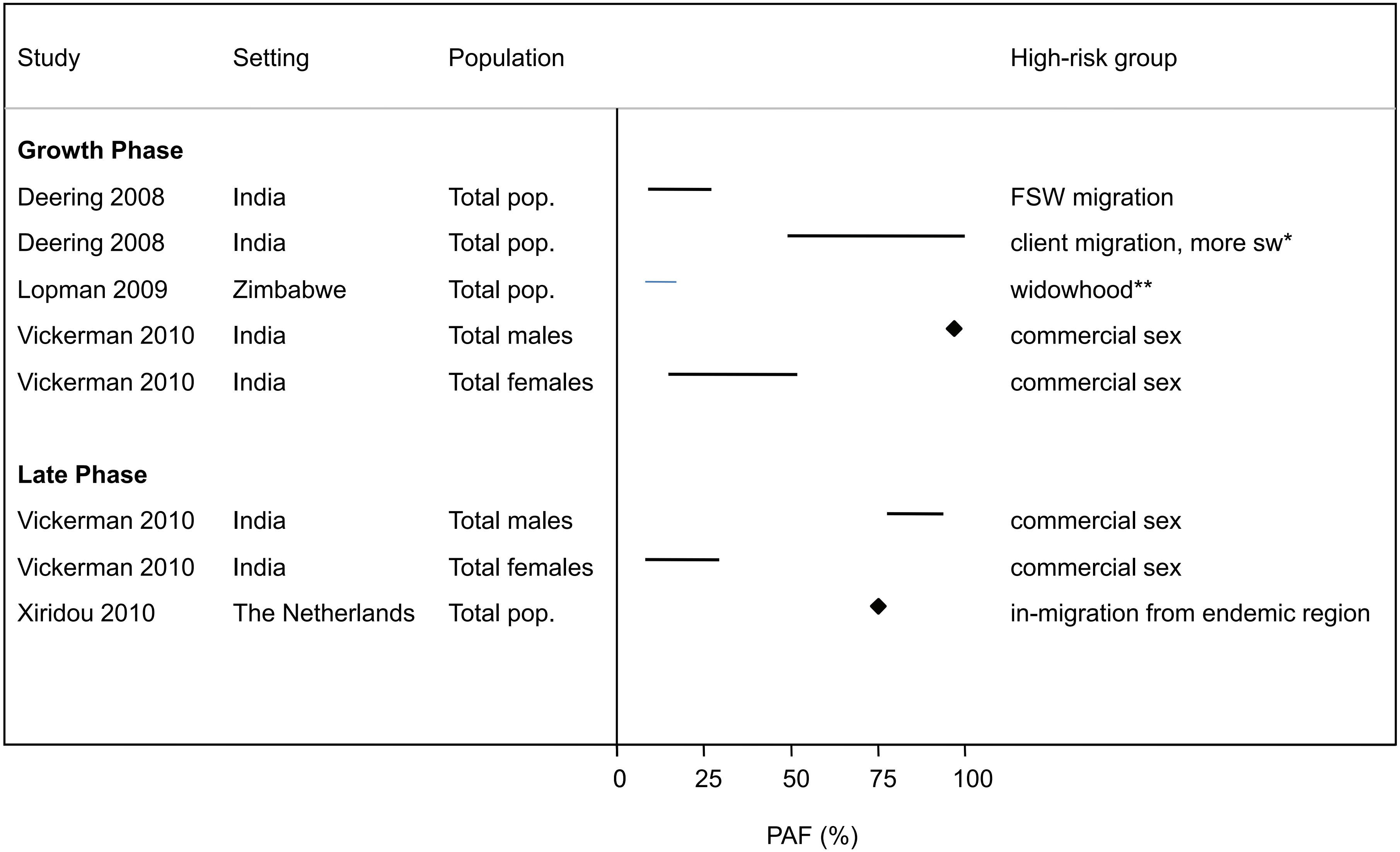

Supplement: Figure S1 — Contribution of high-risk group (HRG) or behaviours to overall HIV transmission. Contribution is measured as the population attributable fraction (PAF, %) of new infections due to a HRG. Study estimates (diamond) and/or the range of within-study estimates are shown for different HRGs and by epidemic phase, and by epidemic size (overall HIV prevalence ≤5% [black], and HIV prevalence >5% [blue]).The PAF was measured in the total population with the exception of Vickerman 2010 [23]. FSW (female sex work) and client migration refers to circular migration. *Client migration associated with more sex work (sw) refers to an increase in the number of local men who pay for sex when migrant clients are away. **Widowhood in the context of a high-prevalence epidemic where HIV prevalence among widowed men and women was 54% and 61%, respectively, and widowed individuals engaged in high-risk sexual partnerships (a larger number of partnerships and preferential mixing with non-widows to preferential mixing with other widows) [28]. (PDF) [file pone.0050691.s001.pdf]
