# Supplementary material for: Impact of High-Risk Sex and Focused Interventions in Heterosexual HIV Epidemics: A Systematic Review of Mathematical Models
Source: PLoS One. 2012 Nov 30;7(11):e50691. doi: 10.1371/journal.pone.0050691 (PMC3511305; doi:10.1371/journal.pone.0050691)

Figure S2

Fraction of HIV infections prevented (prevented fraction, %) following a focused intervention.

Study	Region	Coverage X Efficacy	Time-frame (years)
**Growth phase**			
STI treatment, condoms			
Boily 2002	Benin	≤ 60%	1
Boily 2002	Benin	≤ 60%	10
Boily 2002	Sub-Saharan Africa	≤ 60%	1
Boily 2002	Sub-Saharan Africa	≤ 60%	10
Williams 2006	India	≤ 60%	5
Williams 2006	India	> 60%	5
Williams 2006	India	> 60%	5
Williams 2006	India	≤ 60%	10
STI treatment			
Williams 2006	India	≤ 60%	5
Condoms			
Williams 2006	India	≤ 60%	5
Kakehashi 1998	Japan	> 60%	Equilibrium
**Late phase**			
STI treatment, condoms			
Boily 2002	Benin	≤ 60%	1
Boily 2002	Benin	≤ 60%	10
Condoms			
Deering 2008	India	≤ 60%	11
Vickerman 2010	India	≤ 60%	5
Microbicide			
Vickerman 2006*	Benin	≤ 60%	4
Vickerman 2006*	South Africa	≤ 60%	4
Vaccine			
Hontelez 2011**	South Africa	> 60%	20
Hontelez 2011***	South Africa	> 60%	20
Oral PREP			
Abbas 2007*	Sub-Saharan Africa	≤ 60%	10
Abbas 2007	Sub-Saharan Africa	≤ 60%	10
Abbas 2007*	Sub-Saharan Africa	> 60%	10
Abbas 2007	Sub-Saharan Africa	> 60%	10

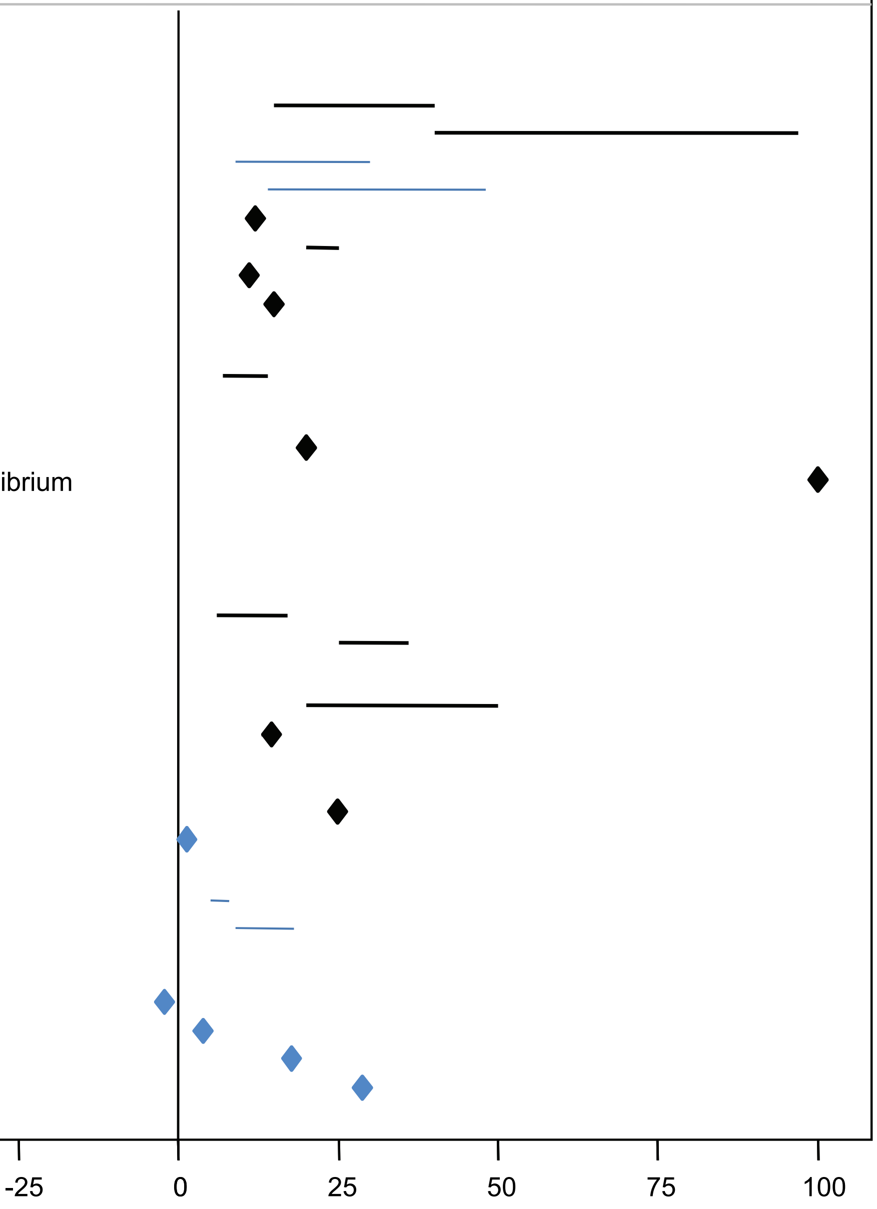

Supplement: Figure S2 — Prevented fraction (%) following a focused intervention. Prevented fraction depicted for various types of intervention, by an aggregate of coverage and efficacy (coverage multiplied by efficacy), and time-horizon for the outcome measurement (years) within studies. Study estimates (diamond) and/or the range of within-study estimates are shown by epidemic size (overall HIV prevalence ≤5% [black], and HIV prevalence >5% [blue]). Efficacy refers to the reduction in HIV susceptibility per sex act (or transmission probability if intervention effect on HIV susceptibility was not differentiated from intervention effect on HIV infectivity). *Risk compensation (Abbas 2007) was modeled as a doubling in the number of partners per year among individuals who received the targeted intervention [39]. *Risk compensation (Vickerman 2006) was modeled as a 5% decrease in baseline condom use (set at 85%) [25]. A vaccine that reduces HIV susceptibility by 78% in the first year with a waning immunity thereafter, repeated every 2 years (**), or every 5 years (***)[22]. STI refers to bacterial sexually transmitted infections. (PDF) [file pone.0050691.s002.pdf]
