# Supplementary figures and images for: Impact of High-Risk Sex and Focused Interventions in Heterosexual HIV Epidemics: A Systematic Review of Mathematical Models
Source: PLoS One. 2012 Nov 30;7(11):e50691. doi: 10.1371/journal.pone.0050691 (PMC3511305; doi:10.1371/journal.pone.0050691)

**Figure S3**

**Number of HIV infections averted per 100,000 adults\*\* following a focused intervention.**

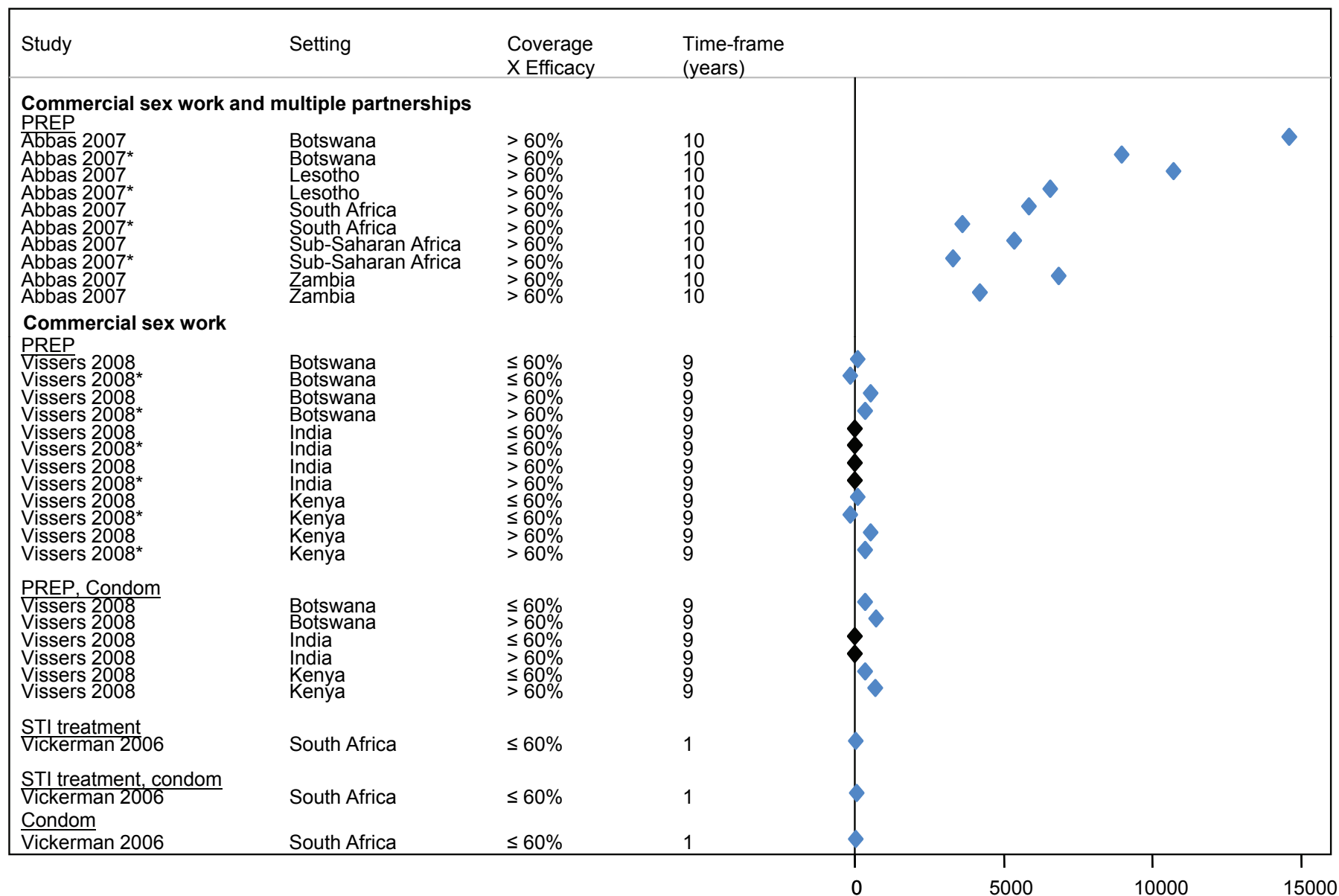

Supplement: Figure S3 — The number of HIV infections averted per 100,000 adults per year after the implementation of a focused intervention. Impact depicted for various types of intervention, by an aggregate of coverage and efficacy (coverage multiplied by efficacy), and time-horizon for the outcome measurement (years) within studies. Study estimates (diamond) and/or the range of within-study estimates are shown by epidemic size (overall HIV prevalence ≤5% [black], and HIV prevalence >5% [blue]). *Presence of risk compensation. Efficacy refers to the reduction in HIV susceptibility per sex act (or transmission probability if intervention effect on HIV susceptibility was not differentiated from intervention effect on HIV infectivity). Commercial sex work includes interventions focused of FSWs or FSWs and clients. The results of a vaccine study are not shown in this forest plot because outcome was measured as infections averted per 100,000 adults who received the intervention [38]. **Vissers 2008 (per 100,000 uninfected adults per year) [36]; Vickerman 2006, Abbas 2007 (per 100,000 adults per year) [26], [39]. STI refers to bacterial sexually transmitted infections. (PDF) [file pone.0050691.s003.pdf]

**Figure S4**

**Relative reduction in overall prevalence following a focused intervention (%)**

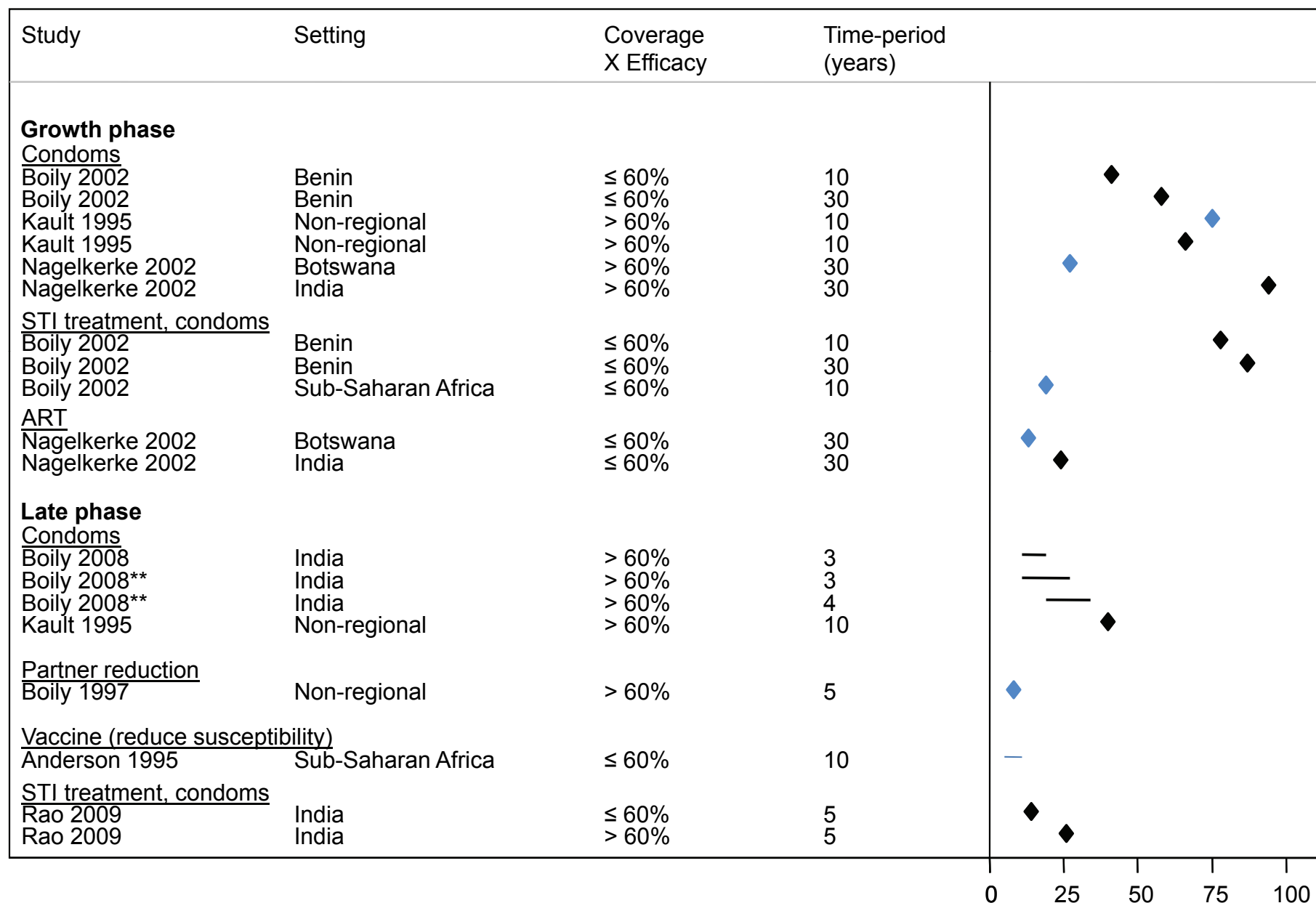

Supplement: Figure S4 — Reduction in prevalence (%) following a focused intervention. Impact depicted for various types of intervention, by an aggregate of coverage and efficacy (coverage multiplied by efficacy), and time-horizon for the outcome measurement (years) within studies. Study estimates (diamond) and/or the range of within-study estimates are shown by epidemic size (overall HIV prevalence ≤5% [black], and HIV prevalence >5% [blue]). Efficacy refers to the reduction in HIV susceptibility per sex act (or transmission probability if intervention effect on HIV susceptibility was not differentiated from intervention effect on HIV infectivity). **Overall prevalence was measured in the antenatal clinic population. STI refers to bacterial sexually transmitted infections. ART refers to combination anti-retroviral treatment. (PDF) [file pone.0050691.s004.pdf]

**Figure S5**

**Relative reduction in overall incidence following a focused intervention (%)**

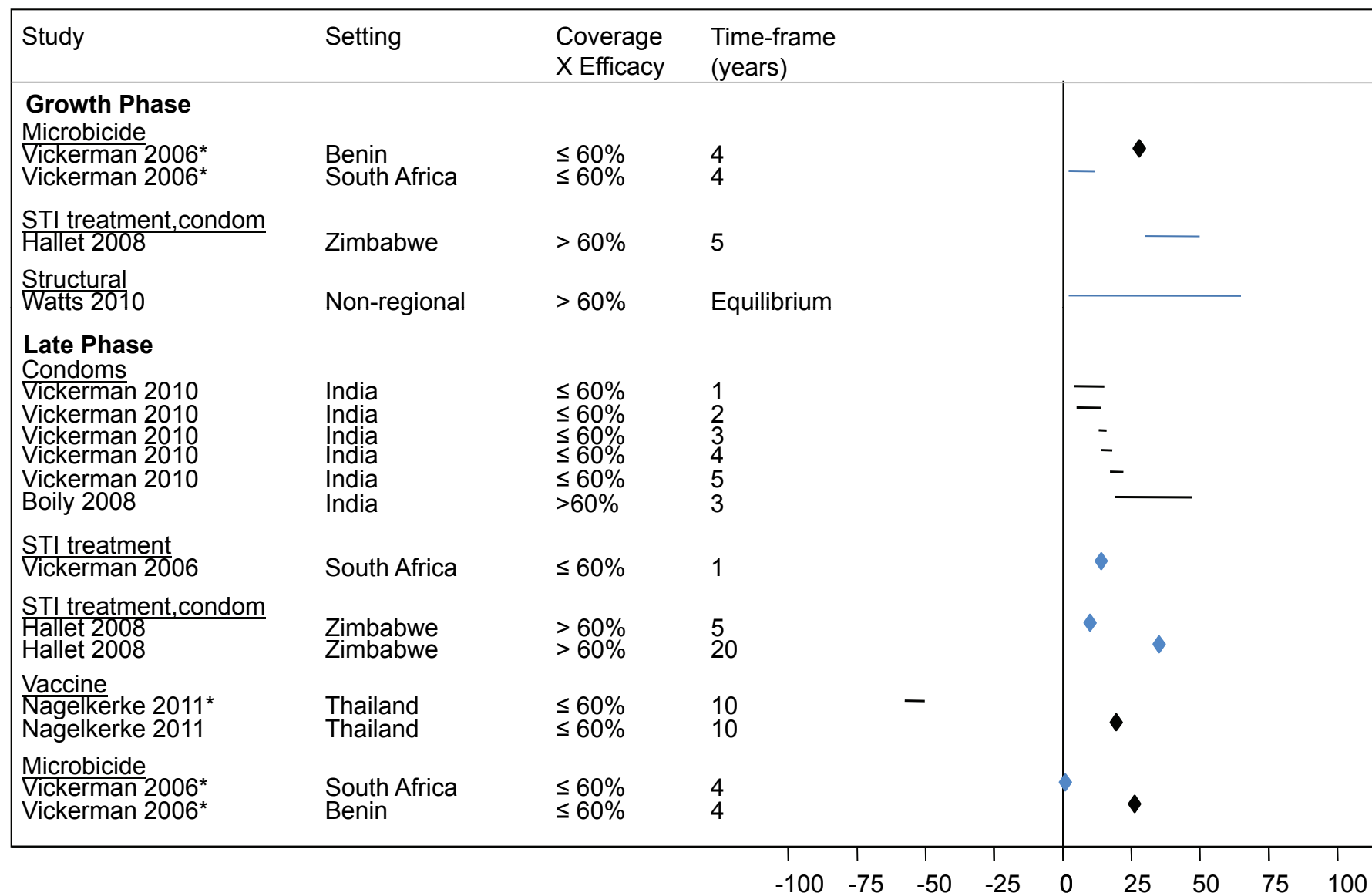

Supplement: Figure S5 — Reduction in incidence (%) following a focused intervention. Impact depicted for various types of intervention, by an aggregate of coverage and efficacy (coverage multiplied by efficacy), and time-horizon for the outcome measurement (years) within studies. Study estimates (diamond) and/or the range of within-study estimates are shown by epidemic size (overall HIV prevalence ≤5% [black], and HIV prevalence >5% [blue]). Efficacy refers to the reduction in HIV susceptibility per sex act (or transmission probability if intervention effect on HIV susceptibility was not differentiated from intervention effect on HIV infectivity). *Risk compensation in Vickerman 2006 was modeled as a decline in condom use from 85% to 80% among those who use the microbicide [25]. Risk compensation (Nagelkerke 2011) modeled as a decline in condom-use from 70% to 50% among those who receive the vaccine [35]. STI refers to bacterial sexually transmitted infections. (PDF) [file pone.0050691.s005.pdf]
