# Supplementary material for: Impact of High-Risk Sex and Focused Interventions in Heterosexual HIV Epidemics: A Systematic Review of Mathematical Models
Source: PLoS One. 2012 Nov 30;7(11):e50691. doi: 10.1371/journal.pone.0050691 (PMC3511305; doi:10.1371/journal.pone.0050691)
Supplement: Table S1 — Summary of contribution of high-risk group or behaviours to overall HIV transmission. (DOC) [file pone.0050691.s006.doc]

**Table S1. Summary of contribution of high-risk group or behaviours to overall HIV transmission.**

| **Setting** | **HRG (or behaviour)** | **Time-frame (years)** | **Cumulative PAF % (population)** | | **Notes** |  |
| --- | --- | --- | --- | --- | --- | --- |
|  |  |  | Growth phase | Late phase |  | Ref. |
| South India | SW | 1 | 95-98 (TP male)  24-42 (TP female) | 86-92 (TP male)  11-28 (TP female) | Mixing: more assortative | [1] |
|  | Circular male migration and sex with HRG at destination | 34-44 | 50-99 (TP) |  | Non-migrant males increased visits to local sex workers when migrants were away. Mixing: more assortative | [2] |
| Zimbabwe | Widowhood | 20 |  | 8 (TP) | Mixing: more assortative | [3] |
|  |  | 20 |  | 10-17 (TP) | Mixing: more disassortative |  |
| The Netherlands | In-migration from regions with higher HIV prevalence and enter local HRG | 1 |  | 22-53 (GP) | Mixing: more proportional | [4] |

HRG (high-risk group); PAF (population attributable fraction, % of all incident infections due to HRG or behaviour); SW (commercial sex work); GP (general population, excluding HRG); TP (total population, including HRG)

References

1. Vickerman P, Foss AM, Pickles M, Deering K, Verma S, et al. (2010) To what extent is the HIV epidemic in southern India driven by commercial sex? A modelling analysis. AIDS 24: 2563-2572. doi:10.1097/QAD.0b013e32833e8663.

2. Deering KN, Vickerman P, Moses S, Ramesh BM, Blanchard JF, et al. (2008) The impact of out-migrants and out-migration on the HIV/AIDS epidemic: a case study from south-west India. AIDS 22 Suppl 5: S165-181. doi:10.1097/01.aids.0000343774.59776.95.

3. Lopman BA, Nyamukapa C, Hallett TB, Mushati P, Spark-du Preez N, et al. (2009) Role of widows in the heterosexual transmission of HIV in Manicaland, Zimbabwe, 1998-2003. Sex Transm Infect 85 Supplement 1: 41-48. doi:10.1136/sti.2008.033043.

4. Xiridou M, van Veen M, Coutinho R, Prins M (2010) Can migrants from high-endemic countries cause new HIV outbreaks among heterosexuals in low-endemic countries? AIDS 24: 2081-2088. doi:10.1097/QAD.0b013e32833a6071.
