# Supplementary material for: Impact of High-Risk Sex and Focused Interventions in Heterosexual HIV Epidemics: A Systematic Review of Mathematical Models
Source: PLoS One. 2012 Nov 30;7(11):e50691. doi: 10.1371/journal.pone.0050691 (PMC3511305; doi:10.1371/journal.pone.0050691)
Supplement: Table S2 — Summary of focused intervention impact. (DOC) [file pone.0050691.s007.doc]

**Table S2a. Summary of focused intervention impact: prevented fraction (% of HIV infections averted).**

| **Setting** | **HRG (or behaviour)** | **Intervention (% efficacy, % coverage)** | **Time-period (years)** | **Prevented fraction % (population)** | | **Notes** | **Ref.** |
| --- | --- | --- | --- | --- | --- | --- | --- |
|  |  |  |  | **Growth phase** | **Late phase** |  |  |
| South India | SW | Generic (20, 100) | 5 |  | 13-16 (TP) | Mixing: more proportional | [1] |
|  | SW | PPT (85,25) or SM(70,75) | 5 | 7-14 (TP) |  | Baseline condom use 69%. Mixing: proportional | [2] |
|  | SW | Condom use (100, 85) | 5 | 20 (TP) |  | Baseline condom use 69%. Mixing: proportional | [2] |
|  | SW | SM (70, 75-90), PPT (85, 25),  Condom use (100, 85) | 5 | 10-12 (TP) |  | Baseline condom use 69%. Mixing: proportional  HIV prevalence in the GP = 2.2% | [3] |
|  | SW | SM (70, 75-90), PPT (85, 25),  Condom use (100, 85) | 5 | 20-25 (TP) |  | Baseline condom use 69%. Mixing: proportional  HIV prevalence in the GP = 0.7% | [2] |
|  | Circular male migration and sex with HRG at destination | Condom use (87, 60) at home and destination | 11 |  | 40-50 (TP) | Baseline condom use 0%, PF larger if migrants increase number of HRG partnerships at destination site and HRG HIV prevalence is higher at destination site. Mixing: more assortative | [4] |
|  | Circular male migration and sex with HRG at destination | Condom use (87, 60) at destination | 11 |  | 20-35 (TP) | Baseline condom use 0%, PF larger if migrants increase number of HRG partnerships at destination site and HRG HIV prevalence is higher at destination site. Mixing: more assortative | [4] |
| Non-regional | SW | Condom use (100, 100) | Long-term |  |  | Epidemic eliminated. Baseline condom use 50%. Mixing: proportional | [5] |
| Benin | SW | SM (100,50), condom use (100,60) | 1 | 20-22 (GP female) | 6 (GP female) | Baseline condom use 50%. Mixing: more assortative | [6] |
|  | SW | SM (100,50), condom use (100,60) | 10 | 58-85 (GP female) | 38 (GP female) | Mixing: more assortative. | [6] |
|  | SW | vaginal microbicide (40, 75) | 4 |  | 24.8 (TP) | Risk compensation: 5% reduction in condom use. Mixing: between proportional and assortative | [7] |
| South Africa | SW | vaginal microbicide (40, 75) | 4 |  | 1.3 (TP) | Risk compensation: 5% reduction in condom use. Mixing: between proportional and assortative | [7] |
|  | SW, multiple partnerships | HIV vaccine repeated every 5 years (78% reduced susceptibility, 0% reduced infectiousness, waning immunity at 6% per month, 100% coverage) | 20 |  | 5-8(TP) | No risk compensation. Mixing: proportional. Lower and upper bounds reflect 30% and 60% vaccine coverage in the GP. | [8] |
|  | SW, multiple partnerships | HIV vaccine repeated every 2 years (78% reduced susceptibility, 0% reduced in infectiousness, waning immunity at 6% per month, 100% coverage) | 20 |  | 9-18(TP) | No risk compensation. Mixing: proportional. Lower and upper bounds reflect 30% and 60% vaccine coverage in the GP. | [8] |
| Sub-Saharan Africa | SW, multiple partnerships | Oral PREP (30,25) | 10 |  | -2.5 to 0.8 | Efficacy against drug-susceptible virus reduced by 100% for drug resistant virus. In the presence of risk compensation (rate of partner change doubles among those receiving PREP), there is a 2.5% increase in the number of new infections over 10 years. Mixing: more assortative | [9] |
|  | SW, multiple partnerships | Oral PREP (60,50) | 10 |  | -1.9 to 6.8 | Efficacy against drug-susceptible virus reduced by 75% for drug resistant virus. In the presence of risk compensation (rate of partner change doubles among those receiving PREP), there is a 1.9% increase in the number of new infections over 10 years. Mixing: more assortative | [9] |
|  | SW, multiple partnerships | Oral PREP (90,75) | 10 |  | 17.7 to 28.8 | Efficacy against drug-susceptible virus reduced by 50% for drug resistant virus. In the presence of risk compensation (rate of partner change doubles among those receiving PREP), the prevented fraction is smaller. Mixing: more assortative | [9] |

STI (sexually transmitted infection), NG (*Neisseria gonorrhoea*), CT (*Chlamydia trachomatis*), PPT (periodic presumptive treatment for STI), SM (syndromic management for STI). SW (commercial sex work – implies intervention targeted to female sex workers [FSW] only unless otherwise stated). Mixing between HRG and wider population; P (mixing is proportional to size of risk groups of the opposite sex), A (mixing is preferential [assortative], such that individuals from one risk group tend to form partnerships with individuals of the same risk group).

**Table S2b. Summary of focused intervention impact: number of HIV infections averted.**

| **Setting** | **HRG (or behaviour)** | **Intervention (% efficacy, % coverage)** | **Time-period (years)** | **Absolute number of HIV infections averted;**  **Late phase only** | **Notes** | **Ref.** |
| --- | --- | --- | --- | --- | --- | --- |
| South Africa | SW | HIV vaccine (30% reduced susceptibility, 84% reduced rate of progression to AIDS, 83% reduced infectiousness, 50% partially protected, 50% fully protected, 10 year duration of protection, 60% coverage), no boosting. | 10 | 0.4-2.4 infections averted per 100 vaccinated | 25% risk compensation. No HIV screening. Mixing: more assortative. | [10] |
|  | SW | HIV vaccine (95% reduced susceptibility, 0% reduced rate of progression to AIDS, 0% reduced infectiousness, 50% partially protected, 50% fully protected, life-long duration of protection, 60% coverage), no boosting. | 10 | 2 additional infections to 3 infections averted per 100 vaccinated | 50% risk compensation. No HIV screening. Mixing: more assortaive. | [10] |
|  | SW | PPT (NG/CT 100 and GUD 50-70, 20) | 1 | 21-36 | Infections per 100,000 adults. Mixing: between proportional and assortative. | [11] |
|  | SW | Condom use (100, 20) | 1 | 10-14 | Infections per 100,000 adults. Mixing: between proportional and assortative. | [11] |
|  | SW | SM (50-79, 20) | 1 | 23-44 | Infections per 100,000 adults. Mixing: between proportional and assortative. | [11] |
|  | SW | PPT (NG/CT 100 and GUD 50-70, 20), condom use (100, 20), SM (50-79, 20) | 1 | 41-65 | Infections per 100,000 adults. Mixing: between proportional and assortative. | [11] |
| India | SW (FSW, FSW + clients) | Oral PREP (50-90, 25-75) | 9 | 0.9-6 | Infections per 100,000 uninfected adults (person-years). No risk compensation. Baseline condom use 90%. Larger impact (upper bounds) if both FSWs and clients are reached, and with higher coverage/efficacy. Mixing: more assortative. | [12] |
|  | SW (FSW, FSW + clients) | Oral PREP (50-90 25-75) | 9 | 16.5 additional infections to 4.4 infections averted. | Infections per 100,000 uninfected adults (person-years). Risk compensation. Baseline condom use 90% declines to 75%. Mixing: more assortative. | [12] |
|  | SW (FSW, FSW + clients) | Oral PREP (50-90, 25-75), condom use (100, 92.5) | 9 | 2.7-6.2 | Infections per 100,000 uninfected adults (person-years). No risk compensation. Baseline condom use 90%. Larger impact (upper bounds) if both FSWs and clients are reached, and with higher coverage/efficacy. Mixing: more assortative. | [12] |
| Kenya, Botswana | SW (FSW, FSW + clients) | Oral PREP (50-90, 25-75) | 9 | 26-785 | Infections per 100,000 uninfected adults (person-years). No risk compensation. Baseline condom use 50%. Larger impact (upper bounds) if both FSWs and clients are reached, and with higher coverage/efficacy. Mixing: more assortative. | [12] |
|  | SW (FSW, FSW + clients) | Oral PREP (50-90, 25-75) | 9 | 236 additional infections to 640 infections averted. | Infections per 100,000 uninfected adults (person-years). Risk compensation. Baseline condom use 50% declines to 35%. Mixing: more assortative. | [12] |
|  | SW (FSW, FSW + clients) | Oral PREP (50-90, 25-75), condom use (100, 62.5) | 9 | 288-909 | Infections per 100,000 uninfected adults (person-years). No risk compensation. Baseline condom use 50%. Larger impact (upper bounds) if both FSWs and clients are reached, and with higher coverage/efficacy. Mixing: more assortative. | [12] |
| Botswana | SW, multiple partnerships | Oral PREP (90,75) | 10 | 8,987-14,617 | Infections per 100,000 adults (person-years). The lower bound represents the estimate with risk compensation (double the number of partners per year if receiving PREP). Efficacy of oral PREP for drug resistant virus is 45%. Mixing: more assortative. | [9] |
| Lesotho | SW, multiple partnerships | Oral PREP (90,75) | 10 | 6,583-10,716 | Infections per 100,000 adults (person-years). The lower bound represents the estimate with risk compensation (double the number of partners per year if receiving PREP). Efficacy of oral PREP for drug resistant virus is 45%. Mixing: more assortative. | [9] |
| Zambia | SW, multiple partnerships | Oral PREP (90,75) | 10 | 4,200-6,838 | Infections per 100,000 adults (person-years). The lower bound represents the estimate with risk compensation (double the number of partners per year if receiving PREP). Efficacy of oral PREP for drug resistant virus is 45%. Mixing: more assortative. | [9] |
| South Africa | SW, multiple partnerships | Oral PREP (90,75) | 10 | 3,601-5,863 | Infections per 100,000 adults (person-years). The lower bound represents the estimate with risk compensation (double the number of partners per year if receiving PREP). Efficacy of oral PREP for drug resistant virus is 45%. Mixing: more assortative. | [9] |
| Southern Sub-Saharan Africa | SW, multiple partnerships | Oral PREP (90,75) | 10 | 3,290-5,356 | Infections per 100,000 adults (person-years). The lower bound represents the estimate with risk compensation (double the number of partners per year if receiving PREP). Efficacy of oral PREP for drug resistant virus is 45%. Mixing: more assortative. | [9] |

STI (sexually transmitted infection), NG (*Neisseria gonorrhoea*), CT (*Chlamydia trachomatis*), PPT (periodic presumptive treatment for STI), SM (syndromic management for STI). SW (commercial sex work – implies intervention targeted to female sex workers [FSW] only unless otherwise stated). Mixing between HRG and wider population; P (mixing is proportional to size of risk groups of the opposite sex), A (mixing is preferential [assortative], such that individuals from one risk group tend to form partnerships with individuals of the same risk group).

**Table S2c. Summary of focused intervention impact: Relative change in HIV prevalence and HIV incidence.**

| **Setting** | **HRG (or behaviour)** | **Intervention (% efficacy, % coverage)** | **Time-period (years)** | **Growth phase** | **Growth phase** | **Notes** | **Ref.** |
| --- | --- | --- | --- | --- | --- | --- | --- |
| **Relative change in prevalence % (population)** | | | | | | | |
| South India | SW | Condom use (90, 80-100) | 3 |  | 11-19↓ (GP female) | Baseline condom use 25-40%. Mixing: more assortative. | [13] |
| India | SW | Condom use (100, 75) | 1-30 | 16-94↓ |  | Baseline condom use 33%. Mixing: proportional. Lower and upper bounds represent impact at 1 year and 30 years, respectively. | [14] |
|  | SW | Combination anti-retroviral treatment (100% reduced infectiousness, 25% per year treatment failure, 50% coverage), life expectancy while on treatment increased by 4 years. | 30 | 24↓ |  | Baseline condom use 33%. Mixing: proportional. | [14] |
| Benin / West Africa | SW | SM (100, 50), condom use (100, 60) | 10 | 19-87 ↓ (GP female) |  | Baseline condom use 50%; greater impact at lower baseline HIV prevalence. Mixing: between proportional and assortative. | [6] |
| Non-regional | SW | Reduce partnership rate per year (clients 20 to 15; FSWs 400 to 50) | 5 |  | 8 ↓ (TP) | Mixing: between proportional and assortative. | [15] |
| Non-regional | Multiple partnerships | Condom use (99, 100) | 10 | 66-75 ↓ (TP) | 40↓ (TP) | Mixing: proportional. | [16] |
| Botswana | SW | Condom use (100, 75) | 1-30 | 4-27↓ (TP) |  | Baseline condom use 33%. Mixing: proportional. Lower and upper bounds represent impact at 1 year and 30 years, respectively. | [14] |
|  | SW | Combination anti-retroviral treatment (100% reduced infectiousness, 25% per year treatment failure, 50% coverage), life expectancy while on treatment increased by 4 years. | 30 | 13↓ (TP) |  | Baseline condom use 33%. Mixing: proportional. | [14] |
| Sub-Saharan Africa | Multiple partnerships | Vaccine (20-50% reduction in susceptibility,60-90) | 10 |  | 5-11↓ | Mixing: more proportional. | [17] |
|  | **Relative change in incidence % (population)** | | | | | | |
| South India | SW | Generic (20,100) | 1-5 |  | 12-22 ↓  (TP) | Mixing: more proportional.  Baseline HIV prevalence 0.9% among GP females | [1] |
|  | SW | Generic (20,100) | 1-5 |  | 9-17 ↓  (TP) | Mixing: more proportional.  Baseline HIV prevalence 1.6% among GP females |  |
|  | SW | Condom use (90, 80-100) | 3-4 |  | 19-47 ↓ (GP) | Baseline condom use 25-40%  Greater impact at 5 years post intervention. Mixing: more assortative. | [13] |
| Thailand | SW | HIV vaccine repeated every 2 years (78% reduced susceptibility, 0% reduced infectiousness, waning immunity at 6% per month, 60% coverage) | 10 |  | 16.4 -22.1 ↓(TP) | No risk compensation. Baseline condom use 70%. Mixing: proportional. | [18] |
|  | SW | HIV vaccine repeated every 2 years (78% reduced susceptibility, 0% reduced infectiousness, waning immunity at 6% per month, 60% coverage) | 10 |  | 50.3 -37.9 ↑ (TP) | Risk compensation (condom-use in commercial partnerships declines from 70% to 50% per sex act). Mixing: proportional. | [18] |
| Zimbabwe | SW | Condom use (100, 80-100), decrease likelihood of bacterial STI by 10% | 5 | 30-50 ↓ (GP) | 10 ↓ (GP) | Baseline condom use 20-45%. Mixing: between proportional and assortative. | [19] |
|  |  |  | 10 |  | 35 ↓ (GP) | Baseline condom use 20-45%. Mixing: proportional. | [19] |
| Benin | SW | vaginal microbicide (40, 75) | 4 | 27-29 ↓ (TP) | 26 ↓ (TP) | Risk compensation (85% condom use decreases by 5%) | [7] |
| South Africa | SW | vaginal microbicide (40, 75) | 4 | 2.2-11.5 ↓ (TP) | 1 ↓ (TP) | Mixing: proportional. | [7] |
|  | SW | PPT (NG/CT 100; GUD 50-70, 20) | 1 |  | 14 ↓ (TP) | Mixing: propotional. | [11] |
| Non-regional | Women who experienced sexual violence | Prevent sexual violence (N/A,100) | Long-term | 5.4-32.4 ↓ (low baseline incidence)  2-65↓ (high baseline incidence) |  | Greater impact when HIV prevalence among violent males > 10% | [20] |

STI (sexually transmitted infection), NG (*Neisseria gonorrhoea*), CT (*Chlamydia trachomatis*), PPT (periodic presumptive treatment for STI), SM (syndromic management for STI). SW (commercial sex work – implies intervention targeted to female sex workers [FSW] only unless otherwise stated). Mixing between HRG and wider population; Proportional (mixing is proportional to size of risk groups of the opposite sex), Assortative (mixing is preferential, such that individuals from one risk group tend to form partnerships with individuals of the same risk group).

**References**

1. Vickerman P, Foss AM, Pickles M, Deering K, Verma S, et al. (2010) To what extent is the HIV epidemic in southern India driven by commercial sex? A modelling analysis. AIDS 24: 2563-2572. doi:10.1097/QAD.0b013e32833e8663.

2. Williams JR, Foss AM, Vickerman P, Watts C, Ramesh BM, et al. (2006) What is the achievable effectiveness of the India AIDS Initiative intervention among female sex workers under target coverage? Model projections from southern India. Sex Transm Infect 82: 372-380.

3. Rao AS, Thomas K, Sudhakar K, Maini PK (2009) HIV/AIDS epidemic in India and predicting the impact of the national response: mathematical modeling and analysis. Math Biosci Eng 6: 779-813.

4. Deering KN, Vickerman P, Moses S, Ramesh BA, Blanchard JF, et al. (2008) The impact of out-migrants and out-migration on the HIV/AIDS epidemic: a case study from south-west India. AIDS 22 Supplement 5: 165-S181.

5. Kakehashi M (1998) A mathematical analysis of the spread of HIV/AIDS in Japan. J Math Appl Med Biol 15: 299-311.

6. Boily MC, Lowndes C, Alary M (2002) The impact of HIV epidemic phases on the effectiveness of core group interventions: insights from mathematical models. SexTransm Infect 78: I78-I90.

7. Vickerman P, Watts C, Delany S, Alary M, Rees H, et al. (2006) The importance of context: model projections on how microbicide impact could be affected by the underlying epidemiologic and behavioral situation in 2 African settings. Sex Transm Dis 33: 397-405. doi:10.1097/01.olq.0000218974.77208.cc.

8. Hontelez JAC, Nagelkerke N, Barnighausen T, Bakker R, Tanser F, et al. (2011) The potential impact of RV144-like vaccines in rural South Africa: A study using the STDSIM microsimulation model. Vaccine 29: 6100-6106. doi:10.1016/j.vaccine.2011.06.059.

9. Abbas UL, Anderson RM, Mellors JW (2007) Potential impact of antiretroviral chemoprophylaxis on HIV-1 transmission in resource-limited settings. PLoS One 2: e875. doi:10.1371/journal.pone.0000875.

10. Johnson LF, Bekker LG, Dorrington RE (2007) HIV/AIDS vaccination in adolescents would be efficient and practical when vaccine supplies are limited. Vaccine 25: 7502-7509. doi:10.1016/j.vaccine.2007.08.047.

11. Vickerman P, Terris-Prestholt F, Delany S, Kumaranayake L, Rees H, et al. (2006) Are targeted HIV prevention activities cost-effective in high prevalence settings? Results from a sexually transmitted infection treatment project for sex workers in Johannesburg, South Africa. Sex Transm Dis 33: S122-132. doi:10.1097/01.olq.0000221351.55097.36.

12. Vissers DC, Voeten HA, Nagelkerke NJ, Habbema JD, de Vlas SJ (2008) The impact of pre-exposure prophylaxis (PrEP) on HIV epidemics in Africa and India: a simulation study. PLoS One 3: e2077. doi:10.1371/journal.pone.0002077.

13. Boily M-C, Pickles M, Vickerman P, Buzdugan R, Isac S, et al. (2008) Using mathematical modelling to investigate the plausibility of attributing observed antenatal clinic declines to a female sex worker intervention in Karnataka state, India. AIDS 22 Supplement 5: 149-164.

14. Nagelkerke N, Jha P, de Vlas S, Korenromp E, Moses S, et al. (2002) Modelling HIV/AIDS epidemics in Botswana and India: impact of interventions to prevent transmission. Bull World Health Org 80: 89 - 96.

15. Boily MC, Masse B (1997) Mathematical models of disease transmission: a precious tool for the study of sexually transmitted diseases. Can J Public Health 88: 255-265.

16. Kault DA (1995) Modelling AIDS reduction strategies. Int J Epidemiol 24: 188-197.

17. Anderson RM, Swinton J, Garnett GP (1995) Potential impact of low efficacy HIV-1 vaccines in populations with high rates of infection. Proc Biol Sci 261: 147-151. doi:10.1098/rspb.1995.0129.

18. Nagelkerke N, Hontelez J, de Vlas S (2011) The potential impact of an HIV vaccine with limited protection on HIV incidence in Thailand: A modeling study. Vaccine 29: 6079-6085. doi:10.1016/j.vaccine.2011.06.048.

19. Hallett TB, Garnett GP, Mupamberiyi Z, Gregson S (2008) Measuring effectiveness in community randomized trials of HIV prevention. Int J Epidemiol 37: 77-87. doi:10.1093/ije/dym232.

20. Watts CH, Foss AM, Hossain M, Zimmerman C, von Simson R, et al. (2010) Sexual violence and conflict in Africa: prevalence and potential impact on HIV incidence. Sex Transm Infect 86 Supplement 3: 93-99. doi:10.1136/sti.2010.044610.
