# Supplementary material for: Impact of High-Risk Sex and Focused Interventions in Heterosexual HIV Epidemics: A Systematic Review of Mathematical Models
Source: PLoS One. 2012 Nov 30;7(11):e50691. doi: 10.1371/journal.pone.0050691 (PMC3511305; doi:10.1371/journal.pone.0050691)
Supplement: Table S3 — Matrix of modeling studies by geographic region, focused intervention, and measured outcomes. (DOC) [file pone.0050691.s008.doc]

**Table S3.Matrix of modeling studies by geographic region, focused** intervention, and measured outcomes.

| **Region** | **Contribution of high-risk group** | **Focused intervention** | | | | | | |
| --- | --- | --- | --- | --- | --- | --- | --- | --- |
|  | **PAF** | **Condoms** | **STI treatment** | **Vaginal microbicide** | **Oral PREP** | **Anti-retroviral treatment** | **HIV vaccine** | **Combination (STI treatment, condom)** |
| South Asia | 2 | 6 | 1 | 1 | 1 | 1 |  | 3 |
| Southeast and East Asia |  |  |  |  |  |  | 1 |  |
| Central Asia |  |  |  |  |  |  |  |  |
| Europe | 1 |  |  |  |  |  |  |  |
| North Africa and Middle East |  |  |  |  |  |  |  |  |
| West Africa |  |  | 1 | 1 |  |  |  | 1 |
| South and East Africa | 1 | 2 | 1 | 1 | 2 | 1 | 3 | 1 |
| Latin America & Caribbean |  |  |  |  |  |  |  |  |
| North America / Australia |  |  |  |  |  |  |  |  |

PAF, population attributable fraction, measured over different periods across studies. STI, sexually transmitted infection. PREP, pre-exposure prophylaxis. Non-regional models are not included in this matrix.
