# Supplementary material for: Impact of High-Risk Sex and Focused Interventions in Heterosexual HIV Epidemics: A Systematic Review of Mathematical Models
Source: PLoS One. 2012 Nov 30;7(11):e50691. doi: 10.1371/journal.pone.0050691 (PMC3511305; doi:10.1371/journal.pone.0050691)
Supplement: Table S4 — Exploratory univariate analysis of the fraction of variance explained by epidemiological and intervention-related assumptions on model estimates. (DOC) [file pone.0050691.s009.doc]

**Table S4. Exploratory univariate analysis of the fraction of variance explained by epidemiological and intervention-related assumptions on model estimates.**

|  | Number of infections averted per 100,000 adults** | Fraction of HIV infections averted (%) | | Relative % reduction in HIV incidence | |
| --- | --- | --- | --- | --- | --- |
| Study assumptions and characteristics | HIV prevalence >5% N_s_=52, N=4 | HIV prevalence ≤5% N_s_=100, N=6 | HIV prevalence >5%  N_s_ =38, N=4 | HIV prevalence ≤5%, N_s_ =20, N=4 | HIV prevalence >5%, N_s_ =36, N=4 |
|  | R^2^ | R^2^ | R^2^ | R^2^ | R^2^ |
| **Epidemiologic characteristics** |  |  |  |  |  |
| Overall HIV prevalence | 0.02 | **0.35** | 0.09 | 0.00 | 0.06 |
| Ratio of HIV prevalence among FSWs to general population females*) | --- | **0.61 (**N_s_=82) | **0.47 (**N_s_=27) | 0.06 | --- |
| Ratio of HIV prevalence among clients to general population males*) | --- | **0.61 (**N_s_=82) | **---** | --- | **0.48 (**N_s_=32) |
| Ratio of number of clients to FSWs | --- | **0.11 (**N_s_=82) | 0.08 **(**N_s_=27) | 0.02 | --- |
| Size of the FSW population (% of total adult females) | --- | **0.14 (**N_s_=82) | 0.08 **(**N_s_=27) | 0.02 | 0.03 **(**N_s_=32) |
| HIV prevalence among FSWs | **---** | 0.08 **(**N_s_=82) | 0.09 **(**N_s_=27) | **0.23** | **---** |
| HIV prevalence among clients | --- | 0.09 **(**N_s_=82) | **---** | --- | **0.25 (**N_s_=32) |
| Risk differential among females: ratio of yearly partner exchange rate (FSWs to general population females) | --- | 0.03 **(**N_s_=82) | **0.24 (**N_s_=27) | 0.04 | 0.03 **(**N_s_=32) |
| Late phase compared with growth phase (reference group) | **---** | 0.09 | 0.09 | 0.01 | 0.01 |
| **Intervention-related characteristics** |  |  |  |  |  |
| Type of prevention tool | 0.03 | 0.08 | 0.07 | 0.04 | 0.04 |
| Intervention coverage of high-risk group | 0.01 | 0.01 | **0.17** | 0.06 | 0.04 |
| Intervention efficacy*** | 0.04 | **0.32** | 0.09 | 0.01 | 0.04 |
| Time-horizon for outcome measurement (years) | 0.02 | 0.09 | 0.01 | **0.18** | 0.00 |
| Risk compensation versus no risk compensation | 0.03 | 0.008 | 0.09 | 0.03 | 0.04 |
| **Type of high-risk group (HRG)** | 0.09 | 0.03 | 0.03 | (all FSWs) | 0.01 |

*If the HIV prevalence in the general population males or females was not given, it was estimated from the total population by subtracting the estimated number of high-risk females or males living with HIV at the time when the intervention was initiated. If time-horizon for outcome measurement was a new endemic equilibrium, the estimates were not included in the analysis [1]. Crude analysis of the fraction of variance explained by each covariate (R^2^), following adjustment for the population in which the outcome was measured (total population, general population, low-risk females, or antenatal clinic patients). **Vissers 2008 (per 100,000 uninfected adults [2]); Vickerman 2006, Abbas 2007 (per 100,000 adults [3][4]); Johnson 2007 (per 100,000 adults vaccinated [5]). N_s_ (Number of scenarios that measured outcome). N (number of studies). ***Efficacy in reducing HIV susceptibility per sex act (or transmission probability if intervention effect on HIV susceptibility was not differentiated from intervention effect on HIV infectivity).

**References**

1. Watts CH, Foss AM, Hossain M, Zimmerman C, von Simson R, et al. (2010) Sexual violence and conflict in Africa: prevalence and potential impact on HIV incidence. Sex Transm Infect 86 Supplement 3: 93-99. doi:10.1136/sti.2010.044610.

2. Vissers DC, Voeten HA, Nagelkerke NJ, Habbema JD, de Vlas SJ (2008) The impact of pre-exposure prophylaxis (PrEP) on HIV epidemics in Africa and India: a simulation study. PLoS One 3: e2077. doi:10.1371/journal.pone.0002077.

3. Vickerman P, Terris-Prestholt F, Delany S, Kumaranayake L, Rees H, et al. (2006) Are targeted HIV prevention activities cost-effective in high prevalence settings? Results from a sexually transmitted infection treatment project for sex workers in Johannesburg, South Africa. Sex Transm Dis 33: S122-132. doi:10.1097/01.olq.0000221351.55097.36.

4. Abbas UL, Anderson RM, Mellors JW (2007) Potential impact of antiretroviral chemoprophylaxis on HIV-1 transmission in resource-limited settings. PLoS One 2: e875. doi:10.1371/journal.pone.0000875.

5. Johnson LF, Bekker LG, Dorrington RE (2007) HIV/AIDS vaccination in adolescents would be efficient and practical when vaccine supplies are limited. Vaccine 25: 7502-7509. doi:10.1016/j.vaccine.2007.08.047.
